# Protein nanofibrils for next generation sustainable water purification

**DOI:** 10.1038/s41467-021-23388-2

**Published:** 2021-05-31

**Authors:** Mohammad Peydayesh, Raffaele Mezzenga

**Affiliations:** 1 ETH Zurich, Department of Health Sciences and Technology, Zurich, Switzerland; 2 ETH Zurich, Department of Materials, Zurich, Switzerland

**Keywords:** Pollution remediation, Environmental impact, Biomaterials - proteins, Organic molecules in materials science

## Abstract

Water scarcity is rapidly spreading across the planet, threatening the population across the five continents and calling for global sustainable solutions. Water reclamation is the most ecological approach for supplying clean drinking water. However, current water purification technologies are seldom sustainable, due to high-energy consumption and negative environmental footprint. Here, we review the cutting-edge technologies based on protein nanofibrils as water purification agents and we highlight the benefits of this green, efficient and affordable solution to alleviate the global water crisis. We discuss the different protein nanofibrils agents available and analyze them in terms of performance, range of applicability and sustainability. We underline the unique opportunity of designing protein nanofibrils for efficient water purification starting from food waste, as well as cattle, agricultural or dairy industry byproducts, allowing simultaneous environmental, economic and social benefits and we present a case analysis, including a detailed life cycle assessment, to establish their sustainable footprint against other common natural-based adsorbents, anticipating a bright future for this water purification approach.

## Abstract (web summary)

Water scarcity is a rapidly spreading global challenge but water purification technologies are often not sustainable. Here, the authors review the research on water purification technologies based on protein nanofibrils as a green and affordable solution to alleviate a water crisis.

## Introduction

**Fig. 1:**
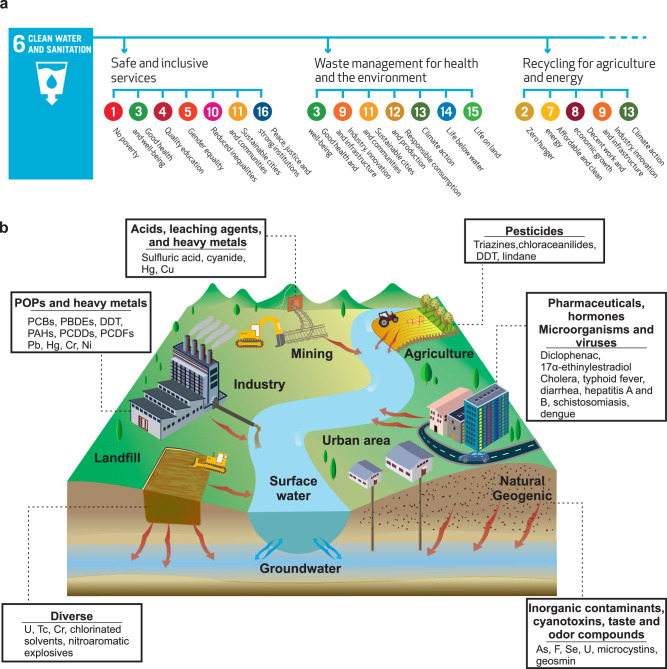
Importance of water and its main sources of pollution. **a** The role of clean water and sanitation (SDG 6) in achieving the other SDGs^2^. **b** Major contaminants of the world water bodies and their sources of release^106^. Panel **a** reproduced with permission from ref. ^2^, Stockholm Environment Institute.

Water is at the center of sustainability and has a pivotal role in ecosystem survival and socioeconomic development. Although water is a basic human right, today one in three humans worldwide does not have access to safe drinking water. Water scarcity is such a global threat to modern society that Clean Water and Sanitation was included as one of the global 17 sustainable development goals (SDGs) for 2030 in the 2015 UN Sustainable Development Summit^1^. SDG6 specifically ensures the availability and sustainable management of water and sanitation for all. Furthermore, its output, i.e., clean water, is an overarching connecting factor for achieving most of the individual SDGs (Fig. 1a)^2^.

Environmental catastrophes, ongoing population growth, and urbanization pose additional threats to a sustainable water management and safe water supply^3^. As observed in Fig. 1b, the major sources of water contamination are the agriculture, landfills, mining activities, industrial, and urban wastewater, by which massive amounts of toxic compounds such as heavy metal ions, organics, and micropollutants are discharged directly into world water resources.

To remediate this tragic situation, various water purification technologies mainly based on distillation, membrane filtration, and adsorption have been proposed^4^. Although these techniques may feature high removal efficiency for contaminants, they rarely can be considered sustainable. To meet the present needs without compromising accessibility to future generations, it is essential to provide sustainable technologies based on an overarching integration of environmental, techno-economic, and social pillars^5^. Removal of contaminants by adsorption is a particularly interesting technology, because it generally features low investment and operating costs, minimal energy requirements and most importantly, it may rely on adsorbing materials extracted from waste or by-products, thus significantly upgrading their position along the value chain, with virtually no environmental footprint^4^.

In this context, proteins occupy a role of prime importance among adsorbing materials for water purification. To start, they are waterborne natural materials exhibiting features of “universal molecular adhesives”^6,7^ and thus they exhibit outstanding capability in binding a wide spectrum of contaminants in water, making them ideal green materials for water purification. Secondly, their specific area can be easily tuned and increased, by converting them, for example, into thin films or nanofibrils of extreme aspect ratios, a key aspect in adsorption processes^8^. Thirdly, and most importantly, proteins can be made promptly available at huge volumes and virtually no cost by recovering or converting them from waste originating from agricultural, cattle, dairy, and food industries, to name only a few sectors^9^. Despite this fact, the value chain of protein-based products is not sustainably managed at the global level if one considers that approximately one-third of the all food production^10^ and 20% of total dairy products^11^ for human consumption are wasted worldwide. Furthermore, many industrial wastes or byproducts contain high amounts of protein such as whey or rapeseed waste cake (including 40% protein)^12^. Valorization of protein waste into the higher-end value products reduces the overall waste streams, adds value to food industry by-products and decreases the overall environmental impact of industry. Thus, revaluing protein-based byproducts and waste into efficient adsorbers and agents for water treatment is not only a viable and green solution for clean water supplying, but also is a significant benefit in waste management and environmental protection.

To this end, we review the current state-of-the-art of protein nanofibrils as an efficient, sustainable, and affordable solution to mitigate the current world water crisis. First, we briefly review the recent history of protein nanofibrils as universal water purification agents since the first proof of concept published in 2016^8^ and we discuss how the field has rapidly expanded to other classes of protein nanofibrils. Then the main classes of protein nanofibrils and their wide-range application in water purification are reviewed. Finally, we discuss the detailed sustainability aspects of the protein nanofibrils for water purification based on the three sustainability pillars and life cycle assessment (LCA).

## Protein nanofibrils as new water purification agents

Protein nanofibrils are a de novo class of nanofibrils materials with unique surface functionality and ultra-high surface-to-volume ratio. They consist of repetitive core sequences, such as hydrophilic and hydrophobic amino acid segments folded within fibrillar and often twisted structures, which are in diameters typically ranging from 5 to 10 nm and length up to several micrometers^13,14^. Protein nanofibrils, are supramolecular polymers rich in β-sheet secondary structures, packed in a cross-β structure with the β-strands perpendicular to the fibril axis in the case of amyloids^14^ and parallel to the fibrils in the case of silk nanofibrils^15^ (Collagen, another class of protein fibrous scaffold based on alpha-helical secondary structure, has not received great attention for water purification, and will not be discussed here). A strong network of hydrogen bonds and van der Waals forces stabilizes their structure^13,14^. Compared to other protein materials, they exhibit outstanding nanomechanical properties and stabilities against enzymatic degradation^14^. In addition to their intrinsic rigidity, they have distinctive features of chirality, polarity, and charge^16^. Based on these remarkable features, protein nanofibrils are emerging scaffolds and dynamic building blocks for engineering smart functional materials for tissue engineering, biomedicine, materials science, nanotechnology, renewable energy, and environmental applications^17^.

Although only recently they have been introduced in the field of water purification^8^, they are rapidly gaining a leading position among sustainable water treatment technologies. Indeed, compared to typical water purification technologies, protein nanofibrils feature many advantages from the sustainability perspective. They have all premises to impact the water purification sector following a fully sustainable, natural-based, low-energy demand, and economically affordable approach^18^. Among different types of protein nanofibrils, amyloid nanofibrils with their multitude of binding sites for adsorbing contaminates have gained a leading role in this context. Different protein sources such as β-lactoglobulin, lysozyme, and bovine serum albumin (BSA) can self-assemble into amyloid nanofibrils under specific conditions^19^. Furthermore, biocompatible silk nanofibrils with superior mechanical stability and flexibility have been widely used in multifunctional composites and filtration membranes.

In this section, we review state-of-the-art research on the four main classes of protein nanofibrils and discuss the main features which make them attractive for sustainable water purification.

### Working principles: adsorption, nano-sieving and their synergistic effects

**Fig. 2:**
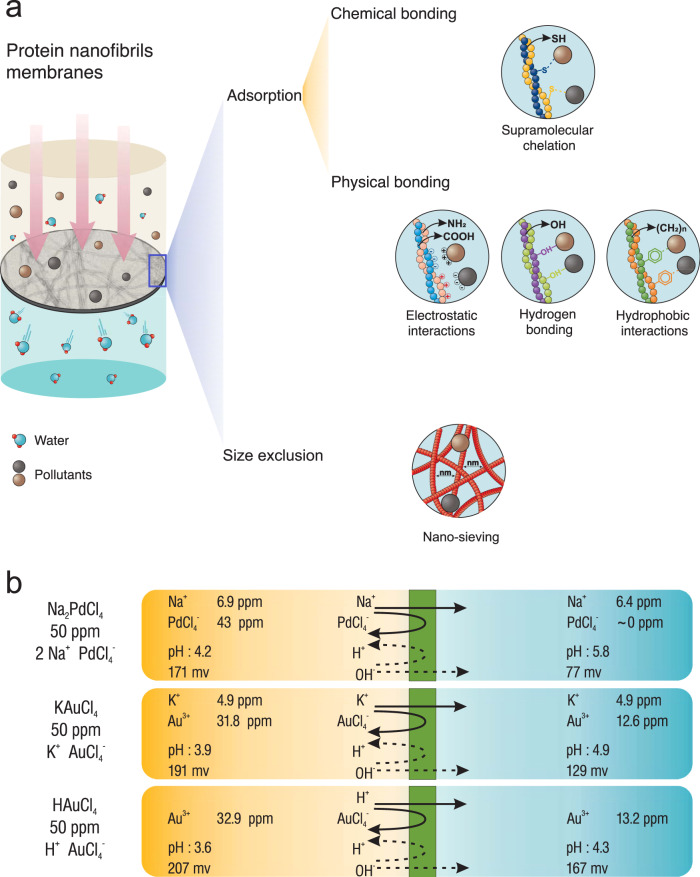
Working principles for water purification by protein nanofibrils for. **a** Different mechanisms for pollutants removal by protein nanofibrils membranes. **b** Selectivity and main parameters during the water filtration by protein nanofibrils membranes.

The principles for pollutants adsorption on the surface of protein nanofibrils are based on both chemical and physical mechanisms, as well as nano-sieving. Amyloid fibrils formation involves protein unfolding, misfolding, aggregation by exposed hydrophobic area, and transition into β-sheet-rich structures^14^. Non-amyloid protein nanofibrils such as silk nanofibrils are also mainly composed of β-sheet^20^. The β-sheets endow the protein nanofibrils with well-defined molecular architecture, good mechanical properties, stability, and high aspect ratios, all essential for water purification applications^21^. The resulting supramolecular structure gives the membranes and adsorbents mechanical robustness and confers high structural stability against most organic solvents, as well acidic and alkaline conditions^14,20–22^. As shown in Fig. 2a, supramolecular chemical chelation between different amino acids available on the protein nanofibrils surface and pollutants, such as heavy metals or organic molecules, plays the main role in the removal of pollutants by protein nanofibrils technologies. During chelation multiple coordination bonds are formed between organic molecules and a single central atom of metal, leading to sequestration of the metal. Chelation, as a typical process in the body, is a major component of enzyme functionality where a metal cofactor such as hemoglobin is involved^23^. The amino acids cysteine, aspartic acid, glutamic acid, and histidine are the most favored motifs to coordinate heavy metals^24^. However, the chelation is not the only involved mechanism for the removal of pollutants by protein nanofibrils: local electrostatic and hydrophobic interactions between the zwitterionic amphiphilic protein nanofibrils and pollutants, as well as hydrogen bonding can also contribute in the binding event^25^. Finally, for finely meshed nanofibril membranes, rejection can also be achieved by size exclusion or nano-sieving, completing the synergistic performance of these materials in water purification^20,26^.

Although protein nanofibrils are efficient in removal of a broad range of contaminants, their specific adsorption capacity for each pollutant is different. To highlight the selectivity of protein nanofibrils membranes, and the changes which the system undergoes during the filtration, important parameters such as ions concentrations and pH are reported in Fig. 2b (unpublished experiments carried out in our laboratory). As observed, the membranes are capable of selectively removing gold and palladium, however, sodium and potassium can pass through the membrane since the supramolecular chelation is highly inefficient for low-atomic number heavy metal ions. This is actually an advantage, since it makes unnecessary post treatment and readjustment of oligoelements mineral composition of water, as in most membrane-based technologies (reverso osmosis, nanofiltration, etc). Since net neutral charge must be preserved, an exchange between the rejected heavy metal ions and H^+^ or OH^−^ occurs in the membrane: in the example shown in Fig. 2b, the rejected heavy metal ions are negative and the permeate is found to contain more electrons (OH^−^) to balance the charge, leading to a higher pH in the permeate. This effect, however, becomes unnoticeable at progressively larger volumes of treated water or for mild levels of contaminants.

Selective adsorption of gold ion (Au^3+^) in the presence of other competitive metal ions, including some common cations in water (Mg^2+^, Cu^2+^, Ni^2+^, and Zn^2+^) and cations in minerals (Sn^2+^, Fe^3+^, Co^2+^, Al^3+^, Cr^3+^, Bi^2+^, Sb^2+^, and Li^+^) by lysozyme amyloid like protein membrane has been investigated^27^. The results reveal that, in the presence of the competitive metal ions at 100 ppm each, the removal rates for gold ions at concentrations from 0.1 to 100 ppm were consistently as high as >90%. Such capability allows the efficient adsorption of Au^3+^ from a mixture of metal ions at a 1–1000-fold higher concentration^27^.

### Manufacturing techniques

Protein nanofibrils, including amyloids and non-amyloids, can be prepared via different methods. Under conditions of low pH values, high temperatures, and several hours of incubation, monomers such as β-lactoglobulin, whey, lysozyme, and BSA can be easily converted to amyloid nanofibrils by unfolding, hydrolysis, and aggregation. For example, amyloid fibrils can be easily prepared from β-lactoglobulin and whey monomers at 90 °C, pH 2, and an incubation time of 5 h. This process converts 75–80% of the entire monomers to amyloid fibrils h^8^. The incubation time for obtaining amyloid fibrils from lysozyme is generally longer, i.e., 24 h^28^. Furthermore, the BSA fibrillation process can be initiated in water and ethanol mixtures at 65 °C^29^. As far as silk nanofibrils goes, there are mainly two methods: exfoliation of silk fibers and self-assembly of silk^15,20,30,31^. Typically, exfoliating natural silk fibers into nanofibrils implies either a chemical (formic acid/CaCl_2_ dissolution) or a physical method (ultrasonication). However, due to the high crystallinity and complex hierarchical structure of silk fibers, both approaches fail to exfoliate silk fibers on the single nanofibers scale and/or yield poor stability for the obtained nanofibrils. To overcome these limitations, recently, a method combining liquid exfoliation, partial dissolution, and ultrasonic dispersion was proposed^30^. This new strategy allows direct exfoliation of silk fibers at the single nanofiber level, while retaining their natural structure and properties^30^. Silk nanofibrils can also be produced directly from the silk aqueous solution through a self-assembly process at 60 °C. Compared to the exfoliation process this process is easier to control and more energetically efficient^31^.

**Fig. 3:**
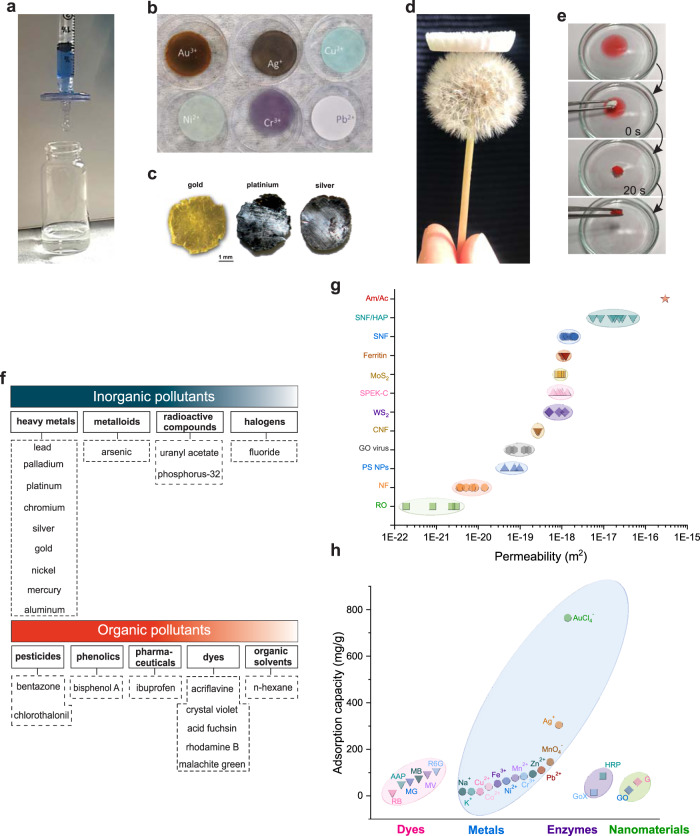
Protein nanofibrils as water purification agents. **a** Image of hybrid silk nanofibrils/hydroxyapatite syringe membrane for the removal of Alcian Blue 8GX from water. **b** Image of hybrid silk nanofibrils/hydroxyapatite membrane after the adsorption of different metal ions. **c** Visual appearance of the elemental precious metals recovered from saturated hybrid membranes based on β-lactoglobulin amyloid fibrils. **d** Image of β-lactoglobulin amyloid fibrils aerogel on top of dandelion flower seeds. **e** A rapid removal of n-hexane (stained with Sudan III) from the water surface by amyloid fibrils aerogel within 20 s. **f** Various water pollutants that so far have been removed from water by amyloid fibrils. **g** Comparison of intrinsic water permeability of hybrid amyloid/activated carbon membranes with the typical commercial NF and RO membranes, as well as the most advanced, recently reported ultrathin membranes (GO graphene oxide, PS NPs polystyrene nanoparticles, CNF carbonaceous nanofiber, SPEK-C sulfonated polyetherketone with cardo groups, SNF silk nanofibrils, HAP hydroxyapatite, Am β-lactoglobulin amyloid fibrils, Ac activated carbon)^22,31,38^. **h** BSA amyloid fibrils film adsorption capacity for dyes (CR Congo red, MG malachite green, RB rhodamine B, MB methylene blue, R6G rhodamine 6G, AAP 4-aminoantipyrine), metal ions, enzymes (HRP horseradish peroxidase, Gox glucose oxidase), and nanomaterials (G graphene, GO graphene oxide)^29^. Panels **a** and **b** reprinted from ref. ^31^. © The Authors, some rights reserved; exclusive licensee American Association for the Advancement of Science. Distributed under a Creative Commons Attribution NonCommercial License 4.0 (CC BY-NC) http://creativecommons.org/licenses/by-nc/4.0/”. Panels **c** reproduced with permission from ref. ^8^, Nature and ref. ^22^, American Chemical Society. Panels **d** and **e** reproduced with permission from ref. ^18^, Wiley.

As observed in Fig. 3, protein nanofibrils have mostly been used in the form of hybrid membranes and aerogels to purify water from heavy metals, metalloids, radioactive wastes, pesticides, dyes, pharmaceuticals, and phenolic compounds^8,18,31,32^.

For fabricating membranes, protein nanofibrils aqueous solution, pure or combined with other materials, is placed on top of a filter support. After drying out the water phase, the remaining thin layer of protein nanofibrils on the top of the support acts as the active layer of membrane. For instance, hybrid amyloid fibrils-activated carbon hybrid membrane can simply be prepared by vacuum-assisted deposition on the surface of a support cellulose filter a homogenous aqueous mixture of β-lactoglobulin fibril (2 wt%) and activated carbon (10 wt%) solutions^22^. The fabrication method is general and extends to other types of protein nanofibril hybrids: for example, amyloid fibrils can be replaced by silk nanofibrils and activated carbon by metallic molybdenum disulfide^33^ to generate diversified organic/inorganic hybrid membranes for water purification.

Furthermore, the extreme aspect ratio of amyloid fibrils can also be used, together with the possibility of tuning their interactions via changes in pH and ionic strength, to allow the formation of hydrogels and aerogels^18^. Water‑stable aerogels from the 2 wt% aqueous amyloid fibrils dispersion were fabricated by freeze‑drying the cross‐linked gel. Amyloid fibrils aerogels could be used for efficient water purification without the use of external pressure, nor induced flow, that is, under perfectly quiescent conditions^18^.

For scaling up and for use in real applications, protein nanofibrils can be modulated in flat-sheet membranes, cartridges, and granules. For example, hybrid membranes can be produced on a large scale by combining amyloid fibrils, activated carbon, and cellulose pulp. Similar to the paper-making process, the water is removed from the mixture by vacuum filtration followed by pressing and drying processes^34^. The prepared membrane sheets can be used in plate and frame filter press units for treating a large volume of contaminated water.

### β-Lactoglobulin and whey amyloid nanofibrils

β-Lactoglobulin is the major protein component of the whey. Whey is a by-product from the dairy industry, which has been long perceived as a food waste, although in the recent decade, a large body of work has been devoted to revalue this protein. Recently, due to their unique functional properties, whey and β-lactoglobulin amyloid fibrils have found interesting industrial applications in microencapsulation, gel formation, as well as foams and emulsions stabilization^35^. By possessing 21 essential amino acids binding sites on their outer surface, β-lactoglobulin and whey amyloid nanofibrils have a great capacity for removing a broad range of contaminants^8^. Furthermore, this technology increases the value of whey protein from waste to a main active ingredient for water purification. The innovation in waste management and environmental preservation along with superior separation performance and low cost, whey amyloid nanofibrils an unchallenged sustainable water purification agent from a sustainability perspective.

In 2016, Bolisetty et al.^8^ introduced this technology by showing the excellent binding performance and capacity of amyloid fibrils on heavy metals removal. Fibrillization process converts monomers, roughly a sphere of 4 nm diameter, into 2 nm thick and several microns long protofilaments, which eventually aggregates further into multistrand and twisted amyloids fibrils. To fully capitalize on binding events between amyloid fibrils and pollutants, hybrid membranes have been fabricated by vacuum filtration of a homogenous aqueous mixture of β-lactoglobulin amyloid fibrils and activated carbon on the surface of cellulose filters^8^. Activated carbon can be used as an ideal matrix, due to its large surface area, endowing high permeability to the hybrid membranes^32^. Then, the as-prepared membranes were used for filtration of contaminated water by heavy metals and radioactive compounds. The heavy metal ions concentration in water dropped by three to five orders of magnitude per passage of filtration^8^. The heavy metals adsorption capacities (i.e., adsorbed mg of heavy metals per g of β-lactoglobulin amyloid fibrils) were extremely high, ranking as lead (996 mg/g) > palladium (366 mg/g) > platinum (235 mg/g) > chromium (149 mg/g) > silver (87 mg/g) > gold (52 mg/g) > nickel (14 mg/g) > mercury (11 mg/g) > aluminum (10 mg/g)^8,22,36^, although it should be noted that the reported adsorption capacities are not static universal values, but rather dynamic relative adsorption capacities, obtained in different experimental conditions for different metals, e.g., using different metal salts, flow, and initial concentrations. Notably, no significant performance decrease appeared over the maximum ten cycles of the filtration. Importantly, the amyloid fibrils hybrid membranes offer the possibility to convert precious heavy metal ions trapped in saturated membranes to elemental metal nanoparticles and films at negligible operating costs. Figure 3c shows visual appearances of the gold, platinum, and silver films, recovered from wastewater by amyloid fibrils membrane and obtained via a simple thermal reduction process^8,22^.

The hybrid amyloid fibrils membranes exhibited high separation performances not only for heavy metals, but also for metalloids^37^. Arsenic as a toxic metalloid exists in the environment and groundwater, mainly in forms of arsenite (arsenic III) and arsenate (arsenic V). In order to evaluate the performance of amyloid fibrils membrane for treating real arsenic contaminated water, samples from Romanian groundwater (386 ppb) and Atitlan Lake, Guatemala (72 ppb) were filtered through the membranes. For both samples, the concentrations after filtration dropped to 1–3 ppb, well below the threshold of 10 ppb for arsenic in drinking water^37^.

Interestingly, the same technology could be applied for the removal of radioactive wastes from water. Uranyl acetate and phosphorus-32 were the selected model pollutants for assessing the performance of amyloid fibrils membranes for water purification from radioactive wastes. The concentrations of uranyl acetate and phosphorus-32 were decreased from 1980 ppm and 12.5 nM to 13 ppm and 0.015 nM, respectively, resulting in a contaminant removal efficiency of three orders of magnitude, or above 99% for both model radioactive pollutants^8^.

To shed light on the exact adsorption capacity of amyloid fibrils for organic contaminants, instead of using hybrid membranes, which contain carbon as a good organic adsorbent, pure amyloid fibrils aerogels were designed and produced^18^. As observed in Fig. 3d, the resulting aerogel was ultralight with low density, high specific area, and high stability in water. The removal of bentazone, bisphenol A, and ibuprofen, as representative water pollutants for pesticides, phenolics, and pharmaceuticals, respectively, was performed via their passive adsorption onto the amyloid fibrils aerogel. The aerogel showed excellent separation performance by removing 92% of bentazone, 78% of bisphenol A, and 98% of ibuprofen from water. In addition, the adsorption capacity of the aerogel for bentazone, bisphenol A, and ibuprofen was 54.2, 50.6, and 69.9 mg/g, respectively. The aerogel was further used for purification of water from dyes and organic solvents. For example, n‑hexane as an organic solvent is completely removed from the surface of water within 20 s by using an amyloid fibrils aerogel (Fig. 3e). The rapid adsorption of organic solvents by amyloid fibrils aerogel is due to the extreme specific area of the aerogels and is driven by the differences in chemical potential between the aerogel host and the surrounding polluted solution. At the end of the adsorption process, the organics loaded aerogels were regenerated with a simple acid washing step, and again reused for removing pollutants. Compared to the fresh aerogel, the regenerated one exhibited identical pollutant removal efficiencies over three subsequent adsorption–regeneration cycles, validating the technology as a sustainable and economically feasible approach for water purification^18^.

Amyloid fibrils can also be combined with other materials for the formation of hybrid nano-adsorbents with high synergistic removal performances. Indeed, while they are efficient water purification agents from heavy metals and organic pollutants, only negligible adsorption capacities are reported for the removal of halogens, such as fluoride. Zhang et al. exploited the high affinity of β-lactoglobulin amyloid fibrils toward highly charged forms of the transition metal zirconium (IV), and achieved the growth and confinement of well-defined zirconium nanoparticles smaller than 10 nm on the outer side-surface of amyloid fibrils. Then, the zirconium-loaded amyloid fibrils nanocolloids were mixed with activated carbon to fabricate hybrid membranes for fluoride removal form water. The obtained membranes showed excellent fluoride binding capacity, superior selectivity against competitive ions, and rapid purification. The membrane removal efficiency exceeds 99% for both low and high fluoride concentration in the feed stream. The ion distribution coefficient (*K*_d_), which is an indicator for the selectivity of the adsorbent, was ~180 times higher than that of typical commercial ion-exchange resins for fluoride removal. The technology was finally validated against fluoride removal by continuous purification of municipal drinking water of a major city in Italy. The results showed that 1750 l of fluoride-contaminated water could be purified per a square meter of the hybrid membranes with removal efficiencies above 99%. Furthermore, the fluoride-saturated membranes could be regenerated and reused several times without any change in performance^32^. Figure 3f summarizes the various water pollutants that have been successfully removed by β-lactoglobulin amyloids. As observed, a wide range of pollutants from different categories can be removed, demonstrating the universal role of this technology in water purification.

Besides the high and broad removal efficiencies, also the very high water permeability of hybrid β-lactoglobulin amyloid nanofibrils membrane distinguishes it from other membrane technologies. In Fig. 3g, the intrinsic water permeability of this membrane is compared with those from the typical commercial NF and RO membranes, as well as those from most advanced, recently reported ultrathin membranes^20,22,38^. As observed, by reaching the value of 2.92 × 10^−16^ m^2^, the water permeability of amyloid–carbon hybrid membrane is at least four orders of magnitude higher than those reported for the typical commercial membranes. Moreover, this permeability is achieved under the transmembrane pressure of only 1 bar, which is not comparable to minimum pressure demands of 10 and 20 bars for NF and RO^22^. Additionally, compared to the other emerging materials for the membrane technology, hybrid protein nanofibrils membranes, including both silk nanofibrils and amyloid fibrils based membranes, exhibit the highest water permeabilities^22,31^. It is important to observe that it is possible to largely reproduce these results directly by using raw materials, such as whey, as a source for amyloid fibrils formation. For example, amyloid fibrils were directly prepared from whey protein isolate and applied to remove chromium and mercury efficiently from water^39^. Additionally, the radionuclides technetium (Tc-99m), iodine (I-123), and gallium (Ga-68) can be removed from radioactively contaminated hospital wastewater with efficiencies above 99.8% in one single step filtration by using hybrid whey amyloid fibrils membranes^34^.

### Lysozyme amyloid nanofibrils

Lysozyme is an enzymatic globular protein, which due to its natural abundance and functional characteristics has gained importance in recent years^40^. The chicken egg white is the most available and accepted source of this protein, as it contains 3.5% lysozyme^41^. Lysozyme is characterized by the presence of 17 positively charged amino acid residues, making the protein mostly positively charged and setting the isoelectric point between 9.5 and 11^40,42^. Owing to its high catalytic and cleaving properties, lysozyme is an important antimicrobial protein, especially for Gram-positive bacteria^41^. Therefore, it is widely used as an additive in the food industry to inhibit the bacteria growth on food materials, enabling long-term storage^41^. Lysozyme plays a key role as a food preservative, by reducing the food products deterioration by maintaining the quality and extending the shelf life of foods^40^. Lysozyme amyloid nanofibrils have been widely used for dyes and heavy metals removal from water, due to their high binding affinity and stability^27,43–45^.

In one study by Morshedi et al.^43^, the ability of pure lysozyme nanofibrils to remove hazardous dyes from water was evaluated against acid red 88, Bismarck brown R, direct violet 51, reactive black 5, and Congo red. By adding protein nanofibrils to the dye solutions, a binding between the nanofibrils and the dyes was observed and after coagulation the solution was cleared by the majority of the dye. The binding mechanism could be attributed to the electrostatic interaction between the ionizable COOH and NH_2_ groups of the nanofibrils and the dyes. Furthermore, intercalation of protein β-sheets and π − π and cationic-π interactions with aromatic residues of dyes may contribute in the binding events. The coagulation efficiency of lysozyme nanofibrils varied between 60 to 98%, based on dyes chemical structure and the other operational conditions, such as pH and temperature. For the range of dyes investigated, most of the efficiencies were higher than the control obtained with activated carbon as a conventional adsorbent for azo dye remediation^43^. Lysozyme nanofibrils can further be functionalized by magnetite nanoparticles to form magnetic nanofibrils for rapid and easy dyes removal for water. Magnetic nanofibrils maintained their high removal efficiency for reactive black 5, acid blue 29, and Victoria blue B dyes after undergoing 20 cycles of adsorption and desorption. After adsorption, the dye-loaded magnetic nanofibrils could simply be collected from water by using an external magnetic field, leaving a clear solution^44^. To ensure that the intermediates formed during protein fibrillization could not have harmful effects on the cell and its mitochondria, the cytotoxicity of water treated with nanofibrils was explored by testing the viability of the cultivated human cell from liver and brain tissues. The results showed no significant difference in the numbers of viable cells between the control and cultivated cells in treated water, confirming the safe water purification achieved by protein nanofibrils^43^.

Lysozyme in its pure form and in the combination with other functional materials, has been widely used for heavy metals removal from water. Cellulose and carbon are the most used matrixes for fabricating lysozyme nanofibrils films and membranes, exploiting their high binding tendency with heavy metals. For instance, dual nanofibrillar-based bio-sorbent films composed of lysozyme and cellulose nanofibrils were fabricated and used for the removal of Hg(II) from water^45^. The obtained freestanding films were flexible and easy to manipulate. The Hg(II) removal efficiency of cellulose/nanofibrils hybrid films, after adding the lysozyme nanofibrils, improved from 35% to above 80%, indicating the high propensity of protein fibrils for adsorbing Hg(II) ions^45^. Furthermore, gold, mercury, lead, and palladium removal efficiencies above 90% were achieved by hybrid membranes composed of lysozyme amyloid fibrils and activated carbon^8^. In real applications of lysozyme nanofibrils for water purification, the cooccurrence of hard cations such as Na^+^, Ca^2+^, and Mg^2+^ may affect the amino acids–heavy metals binding efficiency and results in unsatisfactory separation performance. To resolve this issue, lysozyme nanofibrils can be decorated by grafting various functional species to enhance their selectivity for the target metal in water. For instance, with the aim of fast and robust lead removal from water, lysozyme nanofibrils were conjugated with long-chain polyethyleneimine (PEI) via a green route and using a mussel-inspired polydopamine (PDA) interface coating^46^. The resulting fibrous composites had a high surface-to-volume morphology, which enabled a rapid water purification from lead in 2 min. The lead selectivity index of modified fibrils was ~80 times higher than that of commercial ion-exchange resin 001 × 7, confirming their exceptional selectivity for lead removal from water in the presence of coexisting cations. The continuous application of the adsorbent revealed that by using 1 kg of modified nanofibrils, ~12 m^3^ of lead contaminated water (Pb (II) concentration of 1 ppm) could be purified to the levels below the WHO drinking water threshold value. Furthermore, the exhausted hybrid adsorbent could be easily regenerated and again reused for removing lead with negligible capacity loss^46^. The chemical modifications of lysozyme nanofibrils was also carried out by conjugating ethylenediamine (ED) to improve their water purifying performance for Chromium^47^.

As mentioned above, compared to other proteins, lysozyme is highly positively charged, which gives it a unique antimicrobial ability for water purification. After fibrillization, lysozyme still maintains its antibacterial activity, allowing the design of new hybrid biological/inorganic materials based on lysozyme amyloid nanofibrils. The antibacterial activities assessment of free lysozyme nanofibrils and once immobilized in layered double hydroxides (LDH) against the Gram-positive *S. epidermidis* demonstrated the high efficiency of the protein nanofibrils in water purification from bacteria^48^.

Phase-transitioned lysozyme (PTL) membrane can also be formed by chemical denaturation and fast amyloid-like oligomer protein aggregation at the air/water interface. The obtained freestanding films have a tunable thickness ranging from 30 to 250 nm and pores sizes 1.8 to 3.2 nm, depending on protein concentration. Owing to these properties and differently from the other aforementioned membranes, the separation mechanism in the PTL membranes is primarily size exclusion and not adsorption. The membranes are capable of retaining molecules with the size larger than 3 nm, which leads to excellent separation performance for small molecules. The diffusion rate in lysozyme amyloid-like oligomers membrane was 1–4 orders of magnitude faster than the rate of other conventional filtration membranes, highlighting the potential of this robust and ultrathin membrane for efficient water purification^26^.

### BSA amyloid nanofibrils

BSA, the main protein of plasma, plays a pivotal role in blood as a carrier for many small molecules such as calcium and bilirubin. It also maintains the blood pH and oncotic pressure^49^. BSA is a cheap protein since large quantities are easily derived from bovine blood, a by-product of the cattle industry^50^. Thanks to its biocompatibility, metabolic activity, non-antigenicity, and availability, BSA has gained increasing attention in biotechnology and nanomedicine^51^. As an ampholyte protein (containing both acidic and basic residues), it can bind both cations and anions^52^. By possessing 582 amino acid residues and various functional groups, especially thiols group, BSA is suitable for adsorbing metal ions from water^29,52^.

When processed accordingly, BSA amyloid nanofibrils could form freestanding, biodegradable films through intermolecular interactions. The BSA films obtained with this method are transparent and typically exhibit high toughness and flexibility, allowing them to be engineered precisely into different shapes. The films have high adsorption efficiency for different heavy metals, nanomaterials, dyes, and enzymes, enabling their application in biological water treatment (Fig. 3h). For instance, due to presence of -S-S- and -SH groups in BSA nanofibrils, the film adsorption capacity for gold and silver has been reported to be as high as 765 and 305 mg/g, respectively. Moreover, gold platelets and silver nanoparticles can be reduced within the films, highlighting their performance in recycling precious metals^29^.

BSA can further be combined with various materials to fabricate novel hybrid membranes for water purification. In one promising application, a composite membrane was prepared by simple vacuum filtration of gold nanocluster-decorated BSA nanofibrils and graphene oxide for the detection and removal of mercury from water^53^. While graphene oxide endows the membrane with higher mechanical stability and exchange area, gold nanocluster-decorated BSA nanofibrils play the main role in mercury separation. The removal mechanism is found to be a combination of BSA cysteine residue–Hg^2+^ binding and metallophilic Au^+^–Hg^2+^ interactions. As a result, the hybrid membrane exhibits an efficiency above 90% for removal of mercury from water^53^. Furthermore, hybrid membranes composed of BSA nanofibrils and activated carbon showed removal performances above 90% for gold, mercury, lead, and palladium^8^.

### Non-amyloid protein nanofibrils: Silk nanofibrils

Silk nanofibrils are the fundamental building blocks of silk fibers. Certain insect larvae like *Bombyx mori* produce silk fibers to form cocoons. Over the last few years, the application of silk fibers has gradually been changing from the traditional textiles field to emerging innovative technologies such as tissue engineering, medical sutures, blood-contacting materials, and optical devices^54^. Owing to amino acids such as serine, arginine, and threonine on their side chain, silk fibers are reactive functional filamentous proteins^55^.

Given their biocompatibility and of ease of processing, both silk nanofibrils and fibers have been widely used for designing new composite membranes^31^, hydrogels^56^, and aerogels^57^ for water purification. Furthermore, compared to other protein nanofibrils, silk nanofibrils possess superior mechanical properties and environmental stability, due to the high density of hydrogen bonding and the high values of crystallinity. Although there are many reports on the application of silk fibers composite products for proteins^58^, nanoparticles^59^, dyes^60^ and heavy metals adsorption^57,61,62^, oil–water separation^63^, and antibacterial and antifouling coatings^64^, here, we only focus on silk nanofibrils applications in water purification.

A pure ultrathin silk nanofibrils filtration membrane could be prepared directly by the vacuum filtration of nanofibrils, right after exfoliation from natural *B. mori* (silkworm) silk fibers^20^. The thickness of the membranes can be tuned down to 40 nm with a narrow distribution of pore sizes ranging from 8 to 12 nm. As a result, the membrane could remove efficiently most dyes, proteins, and nanoparticles via molecular sieving and electrostatic interactions. Furthermore, the pure water flux of ultrathin silk nanofibrils membrane, reaching the value of 13,000 L h^−1^ m^−2^ bar^−1^, was more than 1000 times higher than most commercial polymeric membranes such as polysulfone, poly(ether sulfone), and polyamide^20^.

Fabricating multilayer nanoporous membranes based on soft and hard nano-building blocks is a new strategy for achieving optimized separation performance and mechanical stability. In this construction motif, the soft layer with a smaller pore size serves as an active separation layer, while the hard layer with the higher porosity and mechanical resilience acts as a structural support for the soft layer and endows the resultant membrane with higher permeability. Based on this design and by combining protein self-assembly and in situ biomineralization, biomimetic multilayer silk nanofibrils and hydroxyapatite (a calcium-based mineral) nanoplates membrane were proposed as a new hybrid for water purification^31^. First, silk proteins in an aqueous solution were assembled into elongated nanofibrils with diameters around 3 nm and contour lengths of up to 5 mm. Then, the nanofibrils were used as templates for growing needle-like hydroxyapatite nanocrystals. The connected silk nanofibrils networks stabilized the well-formed hydroxyapatite nanoplates in the solution^31^. Finally, to fabricate the multilayer water-insoluble membrane, the dispersion of silk nanofibrils and hydroxyapatite nanoplates was vacuum filtered^31^, following a procedure similar to what used to combine amyloid fibrils and hydroxyapatite nanoplates^65^. The resultant membranes demonstrated high removal efficiencies for dyes, proteins, and colloids (Fig. 3a). As observed in Fig. 3b, the membranes can also be used for efficient removal of heavy metal ions from water. The results revealed that 1 kg of the membrane is capable of removing 164 g gold, 146 g chromium, 137 g copper, and 132 g nickel from the water^31^.

Multilayered inorganic−organic hybrid membranes can also be obtained by hybridizing metallic molybdenum disulfide (MoS_2_), as two-dimensional transition metal dichalcogenide nanosheets, and silk nanofibrils^33^. The composite membrane removal performance can be tuned by altering the component ratios of the two nanomaterials. These membranes were found to efficiently remove six heavy metal ions including lead, mercury, copper, gold, palladium, and silver, as well as six different dyes including rhodamine 6 G, alcian blue 8GX, methylene blue, crystal violet, brilliant blue G, and Congo red. The maximum adsorption capacity of the optimally designed hybrid membrane reaches 759 and 425 mg/g for gold and palladium, respectively. Furthermore, during filtration, the adsorbed precious metal ions were reduced in situ to their elemental nanoparticle form, thanks to the reducing properties of MoS_2_ nanosheets^33^.

In an analogue work inspired by biomineralization, metal–organic frameworks (MOFs) loaded electrospun-silk-nanofibers (ESF) membranes were prepared^66^. The loading process based on in situ growth of MOFs on nanofibrils membrane showed loading rates reaching 36 and 34% for ZIF-8 and ZIF-67, respectively. Compared to typical polymeric membranes and similar works, the MOF loading capacity of ESF membranes was among the highest reported levels. Moreover, the loaded MOFs retained high porosity and surface area, comparable with that of pristine MOFs. The surface area for ZIF-8 and ZIF-67 loaded silk nanofibrils was 562 and 789 m^2^ g^-1^, respectively. By combining the high surface area of MOFs and the adsorption ability of ESF, the final hybrid membrane exhibited excellent separation efficiency for various heavy metal ions and organic pollutants. The removal efficiency of the membrane for arsenic, chromium, rhodamine B, and malachite green was 92, 99, 95, and 99 %, respectively^66^.

Silk nanofibrils can also be hybridized with synthetic polymers nanofibrils to fabricate new membranes. To achieve excellent mechanical stabilities, Kevlar was integrated in regenerated silk materials through fibrillization and hydrothermal treatments^67^. The resultant membrane demonstrated excellent mechanical resilience, two times higher than that of the pristine silk nanofibrils membrane. Furthermore, filtration efficiencies above 96% for dyes (e.g., rhodamine B and Congo red), proteins (e.g., BSA and lysozyme), and gold nanoparticles were found, while the adsorption value for heavy metal ions (e.g., chromium and cobalt) remained below 33%^67^.

A special class of silk fibers is that originating from spiders. Spider silk is also formed by β-sheet rich nanofibrils and it is known as one of the most robust natural materials, with a very high toughness^68^. Beside its outstanding mechanical properties, spider silk also offers an active surface for heavy metal interaction^69,70^. The metal is coordinated by the terminal amino group of spider silk through either carbonyl oxygen or deprotonated amide nitrogen^69^. The interaction of spider silk with metals makes spider webs sensitive environmental bioindicators for airborne metal pollution^71,72^. In water purification, spider silk was used for the batch adsorption of copper and nickel from water^69^. Maximum adsorption capacities of 12.7 and 1.5 mg/g were achieved for copper and lead, respectively. By bioengineering varying repeat units of uranyl binding sequence adopted from a mutated calmodulin sequence, and by relying on self-assembling features of spider silk, new spider silk uranyl-binding proteins were introduced. Selective binding of the uranyl cations from water could be enabled, facilitating uranium collection and removal from the environment^70^.

It should nonetheless be mentioned that the main drawback of using silk products for water purification is their high cost. Compared to foodborne protein nanofibrils, they are significantly more expensive, implying a lower sustainability footprint. In the much broader arena of membranes for water purification, however, their cost remains competitive. For instance, the cost for highly efficient biomimetic multilayer silk nanofibrils and hydroxyapatite nanoplates membrane is ~$3 per gram, which is comparable with that of cellulose nanocrystal materials^31^.

## Sustainability considerations

**Fig. 4:**
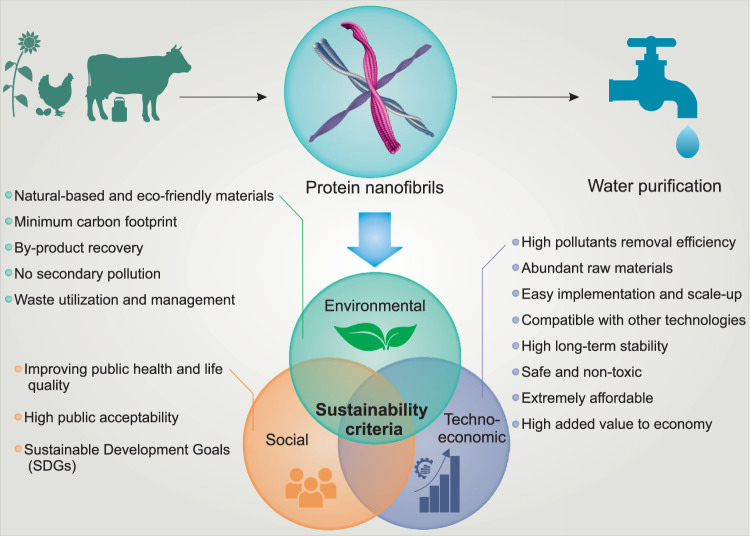
Sustainability aspects of protein nanofibrils water purification agents. The main sustainability impacts of using protein nanofibrils for water purification based on three sustainability pillars of sustainability: Environmental, techno-economic, and social.

Although the removal efficiency in water purification technologies is one of the most important factors, more general sustainability criteria ensuring the technology applicability, ease of use, long-term reliability, and alike, play a significant role and may actually become a critical factor in the implementation of a technology^4,73^. In this section, we highlight the sustainability aspects of the protein nanofibrils for water purification based on the three sustainability pillars^74^ (Fig. 4). These three pillars are environmental, techno-economic, and social, which are more commonly briefly referred to as planet, profits, and people^75^. The detailed sustainability contributions of protein nanofibrils water purification agents in each pillar are presented in Supplementary Table 1.

Environmental sustainability, the first pillar, is defined as the maintenance of world natural capital assets such as water, atmosphere, soil, etc. by reducing and managing the use of resources and assuring that the human’s activities and impacts could not cause harm to the environment. In recent years, the topic has gained increasing attention, and the concept of environmental sustainability has further developed^76,77^. Water purification itself inherently helps achieving environmental sustainability goals by supplying clean water and avoiding the release of contaminants in the environment. However, most of the water purification technologies also have a negative environmental footprint on resources such as soil, air, and energy. Material eco-compatibility and life cycle, secondary pollution, by-product recovery, waste utilization and management, and carbon footprint are the most important criteria for achieving environmentally sustainable water purification technologies^73,74^. Protein nanofibrils are entirely natural-based (milk, egg, bean, silk, etc.), biodegradable, and environmentally friendly. This contrasts positively with the typical membrane water purification technologies, where the membranes are nearly exclusively made by synthetic materials such as polymers, which are not degradable and environmentally friendly. It was estimated that only in 2015 more than 12,000 tons RO modules were discarded, drawing the attention to the magnitude of the nondegradable material disposal problem associated with current water purification technologies^78^. Moreover, during the fabrication of polymeric membranes, many toxic chemicals and solvents are released into the environment, causing further water and soil contaminations^79^. Harmful chemical substances can also be released from the structure of some polymeric membranes, especially under harsh operational conditions and may cause secondary water pollution during the filtration^74^. Unlike other technologies such degradation, which can give rise to secondary pollution by generating toxic metabolites^80^, in each protein nanofibrils technology the pollutants are adsorbed without release of potentially harmful chemical substances into water. Furthermore, as discussed earlier, in recovery applications, adsorbed precious metal ions on protein nanofibrils can be reduced to their elemental forms^8,22,33^, increasing the sustainability value of the technology. Industrial and agricultural waste and byproducts can be used as the source for protein nanofibrils. Whey, for example, is produced at an annual rate of about 121 million tons worldwide^81^, which shows the tremendous potential of this protein waste valorization into the higher-end value products. Waste management by utilizing protein waste streams and minimizing their disposal in the environment is the major advantage of using protein nanofibrils water purification agents. The other important advantage of using the protein nanofibrils technologies compared to other typical water purification technologies is its lower carbon footprint. While the technologies such as distillation and RO rely on sieving, and thus are power and energy-intensive systems, which contribute significantly to carbon dioxide emission^82,83^, protein nanofibrils technologies mostly rely on adsorption and can then operate under gravity-driven filtration, minimizing the energy consumption and greenhouse gases emission.

The second pillar of sustainability, techno-economic, is related to the performance, total costs, and benefits of a technology in achieving a long-term economic growth without negatively affecting the environment and society^84^. The main features of this pillar with respect to water purification technologies are treatment efficiency, stability and reliability, safety, ease of implementation and scale-up, compatibility with other technologies, cost, and impact on economy^73,74^. In the previous section, we discussed in detail about the removal performance, efficiency, and stability of protein nanofibrils water purification agents. As mentioned, compared to the other technologies, they exhibit excellent removal efficiencies for a broad-spectrum of contaminants, including both inorganics and organics, as well as faster binding kinetics and higher flux rates. The technology is reliable, and its performance is stable over the long-term operation and different treatment conditions. Moreover, there is the possibility to easily regenerate the protein nanofibrils and again reuse them in the process without significant changes in the performance, although given the very inexpensive nature of most protein nanofibrils and the side-effects intrinsically related to any regeneration process (e.g., secondary pollution, acid waste management, etc.), protein nanofibrils offer the appealing alternative of direct disposal after use. In all cases, the natural origin of protein nanofibrils makes the technology completely safe and a perfect candidate for household applications of water purification. Protein nanofibrils water purification agents can be modulated in various forms of membranes, cartridges, adsorption columns, granular sedimentation ponds, etc. Their simplicity in operation and maintenance facilitates their wide-range application for water purification from industrial to household levels. To achieve different water treatment plants goals, the protein nanofibrils technologies can be also combined and integrated with other techniques, complying with most water quality requirements boundary conditions. As discussed earlier, not only the sources for protein nanofibrils are natural and affordable, but the purification of waste and byproduct sources can be carried out very inexpensively, which all-together result in low capital cost for the technology. However, consideration should not be limited only to the capital cost, including procurement, installation, and commissioning but also to the operating and entire lifecycle expenses. And in this particular aspect, operating costs of the protein nanofibrils technologies are minimal among all water purification technologies, as they require virtually no energy, functioning for example solely by vacuum filtration and/or gravity driving forces.

The last (but not least) pillar of sustainability is social, directly emphasizing on people, their rights and life quality^85^. The public acceptance of a technology, its role in improvement of public health and life quality as well as in achieving sustainably any of the development goals are the most important criteria for considering a water purification technology as socially sustainable^73,74^. The possibility of purifying water at minimum price and with the smallest environmental footprint by using food waste as precursors, makes protein nanofibrils an outstanding technology from public acceptance perspective. Any protein nanofibrils technology can ideally fulfill the main SDGs by, for example, sustainable management of water and sanitation for all (SDG6), by contributing to ending poverty in all its forms (SDG1), mitigating hunger, achieving food security and improving nutrition and promoting sustainable agriculture (SDG 2) and by ensuring healthy lives and promoting well-being for each individual (SDG 3). Furthermore, the technology contributes in reducing inequalities (SDGs 4, 5, and 10), making sustainable cities and communities (SDG 11), ensuring sustainable consumption and production (SDG 12), saving the environment and climate (SDGs 13, 14, and 15), and promoting peaceful societies for sustainable development worldwide (SDG 16)^86^.

**Fig. 5:**
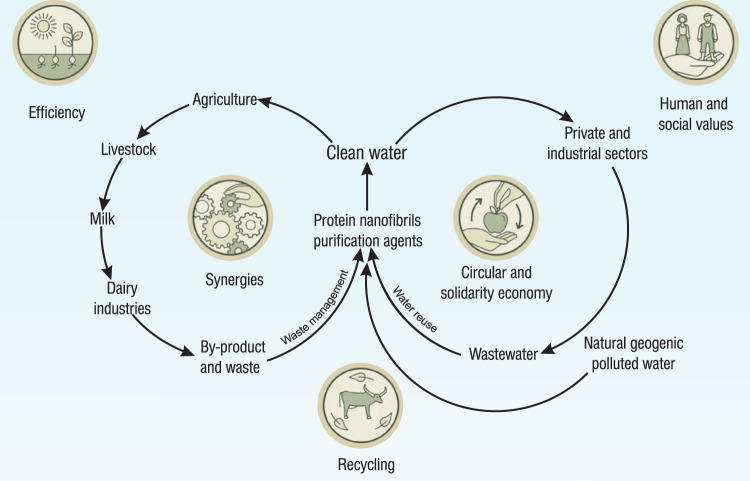
Role of protein nanofibrils in circular economy and their impact on Agroecology. Individual visual elements/medallions of agroecology reproduced with permission from ref. ^87^, Food and Agriculture Organization of the United Nations.

The role and impact of protein nanofibrils purification agents in circular economy, as well as agroecology is represented in two integrated cycles of waste management and water reuse as seen in Fig. 5.

The major application of clean water can be divided into two independent cycles: the first one being the agricultural cycle and the second one the private and industrial sectors. Although both cycles profit from using the clean water source, they produce contamination, directly affecting the water quality. Additionally, agriculture can result in production of waste and by-products, which can be further valorized. Managing waste and recycling water by utilizing the technology of protein nanofibrils purification leads to the interconnection of the two cycles and the purified water can be used further in both processes. Protein nanofibrils purification enhances the efficiency of both cycles greatly through minimizing the dependence on external resources. Shifting the focus on the use of naturally available resources proves to be more step-economical. Waste does not occur naturally in ecosystems and is a man-made concept. Therefore, recycling natural resources decreases waste production and pollution, which in turn further boosts the efficiency of the cycles. Additionally, the increased utilization of natural resources stimulates the generation of new sources of synergies, which improves the ecological functions in agriculture. Generally, using protein nanofibrils purification is an environmentally conscious and innovative method to combine water usage efficiently in different sectors, enabling the social well-being of current communities and also those of future generations.

From the ten elements of agroecology, five are directly affected by the incorporation of protein nanofibrils into the two cycles: circular and solidarity economy, synergies, recycling, efficiency, and human and social values^87^. Incorporating protein nanofibrils into the circular economy allows sustainable markets to be established and to flourish, from which both producers and consumers of the food industry can profit. Global wastewater and by-product challenges can be successfully tackled, while persevering the local environment and its resources. By reusing waste products, new synergies are created via waste management and water treatment. Purification with protein nanofibrils recycles contaminated water within the agricultural, private, and industrial sectors. Through recycling, the total amount of waste, as well as the usage of external resources decreases, leading to a greater efficiency of the consumed water within the two cycles. Natural resources are used more intensively with the integration of protein nanofibrils in the agroecological system. This simultaneously reduces cost, as well as potential exhaustion of the local environment from overusing external resources and its dependency thereon. Utilizing protein nanofibrils as purification agents is a sustainable and low-cost solution, thus, providing a water purification technology that is inclusive for communities of all levels of income.

Table 1 places the technology of water purification by protein nanofibrils in a wider context, by comparing and benchmarking it against the two other common natural-based adsorbents, activated carbon, and nanocellulose; in the three cases, advantages and disadvantages are critically discussed.

**Table 1:** Advantages and disadvantages of protein nanofibrils in water purification compared to activated carbon and nanocellulose.

Adsorbent	Advantages	Disadvantages
Activated carbon	High removal efficiency for organics^90^ Simple preparation^4^ Easy maintenance^4^ Low cost^90^	Moderate removal efficiency for inorganics^4^ Production from nonrenewable coal^88^ Energy-intensive thermal activation^88^ High emissions of CO_2_ equivalents during preparation and activation^89^ Not effective against pathogenic bacteria and viruses, and can harbor bacteria, leading to bacterial growth^91^ Poor selectivity due to nonspecific adsorption mechanisms^90^ Short service life^4^
Nanocellulose	The high removal efficiency of surface-modified nanocellulose for both organics and inorganics^92,93^ Biobased from abundant sources^92,93^ High mechanical stability and processability^92,93^ Acceptable performance efficiency after regeneration^104^	Moderate removal efficiency of pure nanocellulose^92,93^ High cost for production and modification^93^ Prone to biological degradation^92^ Strong tendency to self-aggregate through hydrogen bonding, reducing their available adsorption sites^92^ Little data on removal of multiple types of coexisting pollutants^92^
Protein nanofibrils	Excellent removal efficiency for both inorganics and organics^8,18^ Biobased from affordable sources^34^ Simple preparation^22^ Low cost (e.g., by using whey or other raw vegetable proteins)^34^ High service life^8,22^	Moderately low selectivity^8,27^ Possible microorganism contamination^49^ Little data on regeneration of the adsorbent^18^ Possible high cost (in the case of silk nanofibrils, particularly spider silk)^105^

Adsorption via activated carbon is a tertiary wastewater treatment that has exhibited lower environmental impacts than other purification strategies, such as reverse osmosis and ozone/ultraviolet light oxidation. However, it still has a significant impact on the environment since activated carbon is typically produced from nonrenewable coal and requires energy-intensive thermal activation^88^, particularly the high emissions of CO_2_ equivalents, which amounts to 11 times the final mass product^89^. Activated carbon has high adsorption capacities for organic pollutants; however, its efficiency for removing inorganic pollutants, bacteria, and viruses is limited^4,90,91^.

Nanocellulose, including cellulose nanocrystals (CNCs), cellulose nanofibrils (CNFs), and bacterial cellulose (BC), is a new class of biobased adsorbents with promising potential applications in water purification^92^. CNF, compared to CNC, is mainly used in the works related to water purification. However, surface functionalization is a crucial factor in nanocellulose-based adsorbents to boost the adsorption capacity and selectivity for a specific class of pollutants^92^. Although cellulose itself is the prevalent polymer on earth, nanocellulose production and surface modification processes are not still cost-efficient, and their scale-up for industrial production is still limited to the pilot scale^92,93^.

To provide a more quantitative insight of the environmental impact of protein nanofibrils, activated carbon, and nanocellulose for water purification we perform a LCA in the three cases. The evaluation was performed using the protocol ISO 14040/44 standard, as an attributional and prospective LCA for an emerging product, similar in scope to the studies by Walser et al.^94^ and Arvidsson et al.^95^. The present study compares the environmental impact of the functionalization of these materials in a dynamic adsorption process for treating 10 m^3^ of lead-polluted water with a concentration of 100 ppb (LCA functional unit: removal of 1 kg lead). The LCA assesses cradle-to-use life cycle impacts of the adsorbents and assumes the water treatment process and waste management of these bio-adsorbents to be common and thus not included in the assessment^96^. The dynamic lead adsorption capacities of protein nanofibrils, nanocellulose, and activated carbon are 995.7^8^, 65^97^, and 1.1 mg/g^8^, respectively. Therefore, for the LCA goal of removing 1 kg lead from water, 1, 15.4, and 909.1 kg of protein nanofibrils, nanocellulose, and activated carbon, respectively, are needed.

Life cycle inventory (LCI) for all adsorbents was performed and summarized in Supplementary Table 2. For protein nanofibrils, the process data were provided directly by our laboratory experiments for whey amyloid fibrils. For nanocellulose, the inventory data for TEMPO-oxidation with homogenization preparation route, which has the lowest environmental impact compared to the other preparation processes, was used based on the LCA assessed by Li et al.^98^. The source of inventory data for all input materials and energy was the Ecoinvent 3 database. SimaPro v. 8.3 was used to build life cycle models. To evaluate the impact of the LCI, the ReCiPe midpoint (H) method was used to cover a broad range of impact categories, 18 in this case. Furthermore, the energy use was calculated as cumulative energy demand (CED), measured in GJ.

**Fig. 6:**
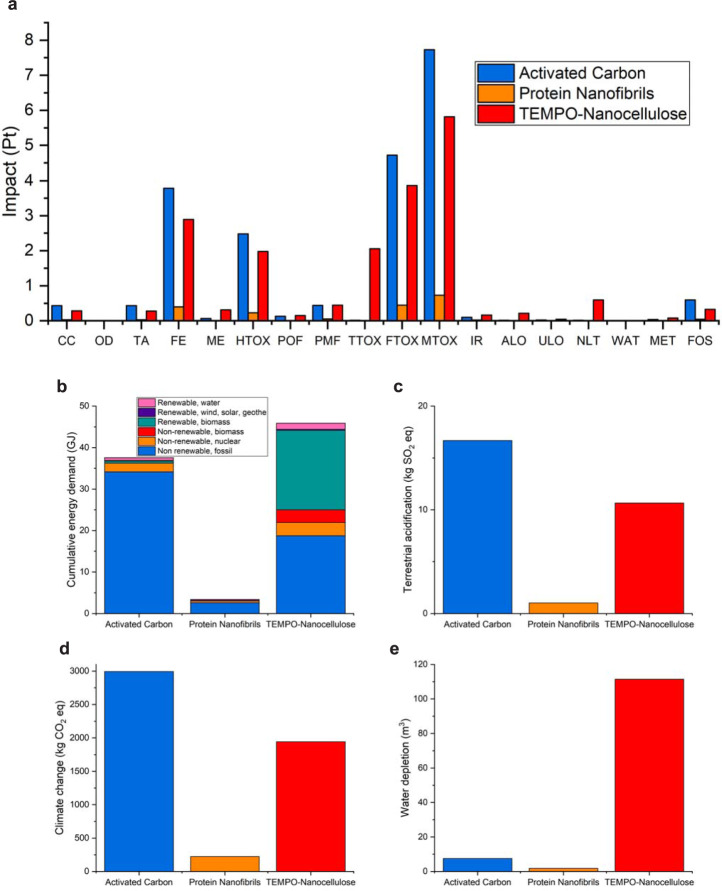
LCA of Protein nanofibrils compared to nanocellulose and activated carbon to remove lead from water. **a** Normalized environmental impact profile of application of protein nanofibrils, nanocellulose, and activated carbon for purifying water from lead, comprising all 18 impact categories of the ReciPe method (CC climate change, OD ozone depletion, TA terrestrial acidification, FE freshwater eutrophication, ME marine eutrophication, HTOX human toxicity, POF photochemical oxidant formation, PMF particulate matter formation, TTOX terrestrial ecotoxicity, FTOX freshwater ecotoxicity, MTOX marine ecotoxicity, IR ionizing radiation, ALO agricultural land occupation, ULO urban land occupation, NLT natural land transformation, WAT water depletion, MET metal depletion, and FOS fossil depletion). **b** LCA results comparison based on cumulative energy demand impact. **c** LCA results comparison based on terrestrial acidification impact. **d** LCA results comparison based on climate change impact. **e** LCA results comparison based on water depletion impact.

The normalized environmental impact profile of applying protein nanofibrils, nanocellulose, and activated carbon for purifying water from lead, comprising all 18 impacts, is presented in Fig. 6a (the exact amount of each category impact is summarized in Supplementary Table 3).

As observed, freshwater eutrophication, human toxicity, and freshwater and marine ecotoxicities have the highest relative contribution to the environmental impacts. Using protein nanofibrils for water purification results, almost in all categories, in lower environmental impact than nanocellulose and activated carbon. This superiority is due to their high adsorption capacities (less material required) combined to a greener and simpler production process. CED is a key impact category, which correlates with the other environmental impacts and can be further classified into the categories of nonrenewable (fossil, nuclear, and non-renewable biomass) and renewable (renewable biomass, solar/wind/geothermal, and hydro) energy^98^. In Fig. 6b, the higher energy demand for the production of required amounts of nanocellulose and activated carbon compared to the protein nanofibrils can be observed. The high energy need for nanocellulose production and treatment has been described as the “Achilles’ heel” of CNF^99^. On the other hand, the production of activated carbons suffers from its high energy demand on fossil fuels as a nonrenewable energy source. The LCA comparison based on other crucial impact categories, i.e., terrestrial acidification, climate change, and water depletion, is presented in Fig. 6c–e. As observed in Fig. 6c, the minimum changes in soil chemical properties following the deposition of nutrients in acidifying forms (reflected by kg SO_2_ eq) occur using protein nanofibrils. While the application of activated carbon and nanocellulose for removing 1 kg lead from the water approximately results in 3000, 2000 kg CO_2_ eq, by using protein nanofibrils only 200 kg CO_2_ eq are emitted, one order of magnitude less (Fig. 6d). Furthermore, as reflected in Fig. 6e, compared to the other two adsorbents, nanocellulose has the highest adverse impact on water resources, mainly due to the release of many chemicals into water during its production and treatment, as well as its high energy demand. Altogether, the LCA undoubtedly points at protein nanofibrils as a more environmentally friendly adsorbent over nanocellulose and activated carbon for the representative water purification case study considered.

Box 1 In this section, via a separate box, we also compare the sustainability footprint of protein nanofibrils with the two other common natural-based adsorbents: activated carbon and nanocellulose. The comparison is performed against water purification from both inorganics and organics contaminants. The sustainability aspects of the technologies are considered by emphasizing the advantages in terms of eight discriminants involved in the use of these technologies, along the lines of our recent analysis for sustainable technologies in water purification from heavy metals^4^, yet in a much simpler and case-specific approach: environmental friendliness, stability, selectivity, simplicity of preparation, public acceptability, cost, and removal efficiencies for inorganics and organics. The adsorbent performance against each discriminant is assessed on a three-rank basis and ranked as a low (*i* = 1), medium (*i* = 2), and high (*i* = 3) level score. An overall sustainability footprint is then estimated for both technologies by summing up the individual components as: $100 \% \cdot \mathop{\sum}\limits_{j=1}^{8}\,{(\frac{i}{3})}_{j}\cdot \frac{1}{8}\,$, where each of the *j* = 8 discriminant carries a weight between 1/3 and 1 depending on the score *i*. As observed in Box 1, compared to other adsorbents, activated carbon is the least sustainable adsorbent, particularly due to its high adverse environmental impacts^89^ and the low adsorption efficiencies for inorganics such as heavy metals^4^. Nanocellulose scores medium in both factors; yet, the challenges related to cost-effective upscaling and limitations in specificity are reflected in the final score^93^. Overall, this comparative analysis reveals the superiority of protein nanofibrils over activated carbon and nanocellulose in terms of their sustainability footprint for water purification.

Sustainability footprint comparison between protein nanofibrils, activated carbon, and nanocellulose as the water purification agents. The characteristics considered are environmental friendliness, stability, selectivity, simplicity of preparation, public acceptability, cost, and removal efficiencies for inorganics and organics. The performance of the adsorbents in each parameter is evaluated on a three-rank basis of low (red), medium (yellow), and high (green) levels. The overall sustainability footprint of each adsorbent is a weighted average of the score in each characteristic (see text for details).

## Conclusions and perspectives

The emerging technologies of protein nanofibrils for water purification embody the concept itself of sustainable development. By contributing to delivering clean water, reducing worldwide food waste, improving global health, minimizing energy consumption with virtually no CO_2_ emission, and minimal impact on climate changes, protein nanofibrils water purification agents align with many of the SDGs set forward by the United Nation Resolution 70/1 for the 2030 Agenda.

Despite the many advantages discussed above for protein nanofibrils based water purification technologies, there still is a need to further expand this field towards vegetable proteins originating from agricultural waste, as the possibility to generate protein nanofibrils from soy, pea, rice, potatoes, corn, etc. is already demonstrated (for a list of vegetable proteins to be discussed see, for example, Cao & Mezzenga Adv- Coll. Interf. Sci. 2019)^9^, yet underused in the field of water purification.

One of the disadvantages of adsorption and membrane technologies, in general, is the disposal of contamination-saturated adsorbents and membranes after the water purification process. Compared to the other adsorbents, protein nanofibrils do allow for improvement to some extent. For instance, one-step adsorption and reduction of hazardous metal ions to their elemental states on the protein nanofibrils surface is an efficient approach to reduce their exposure to the environment as well as their toxicity. Various amino acids endow protein nanofibrils with a reducing ability for converting toxic heavy metal ions to their elemental form. For example, lysozyme amyloid fibrils contain tryptophan (Trp), histidine (His), aspartic acid (Asp), and tyrosine (Tyr) residues, which have the potential to reduce gold ions to their elemental form. The imidazole, indolyl, and carboxyl moieties of these amino acids are further functional groups for reducing heavy metals. Once elemental metal nanoparticles are formed on the membrane surface, they are rapidly stabilized by cysteine (Cys) residues in protein nanofibrils through strong metal-thiol coordination^27^ and can then be readily separated and recovered. Similarly, gold and silver ions were converted to gold platelets and silver nanoparticles within the BSA nanofibrils films^29^. While these reports clearly demonstrate the protein nanofibrils’ potential for recycling heavy metals and mitigating heavy metal toxicity to the environment, degradation of organic pollutants still need to be demonstrated via this technology. Since the incineration of the organic waste results in carbon dioxide production, the hybridization of protein nanofibrils with photocatalysts for the degradation of organic pollutants can be considered a greener approach.

For improving the selectivity or targeting the adsorption of specific pollutants, protein nanofibrils can be further engineered and functionalized. The functional groups on the protein nanofibrils surface, such as amine, carboxyl, hydroxyl, and thiol, can be substituted with other desirable functional groups through a surface modification process. For instance, for enhancing the selectivity for gold and palladium, α-cyclodextrin, benzimidazole, crown ethers, or sulfur-containing ligands, respectively, can be grafted to nanofibril proteins. Additionally, depending on the specific adsorption, interfering sites from protein nanofibrils can be eliminated by methylation of amine groups, acetylation of amine and hydroxyl groups, and esterification of carboxyl groups^27^.

Microorganism contamination, biofilm formation, and eventually fouling are among the major challenges for the long-term application of protein nanofibrils in water purification. Microorganisms such as bacteria can attach to the proteins and subsequently damage and destroy them. A dense matrix of these microorganisms can cause biofilm formation on the surface and fouling. Although fouling is a typical phenomenon in membrane process technologies, its occurrence on protein surfaces is more likely. Several solutions have already been advanced in the field of protein nanofibrils. For example, it was reported that BSA in amyloid oligomers could be used to fabricate antifouling coating, films, and membranes^49^. Robust and freestanding amyloid-like nanofilms were fabricated based on fast protein aggregation via the rapid reduction of intramolecular disulfide bonds of BSA. The obtained films were found to suppress the biofilm formation of a broad range of bacteria and fungi. The antifouling properties of BSA amyloid-like are related to the balance of positive and negative charges on the albumin protein surface. However, no evident antifouling capacity was observed for the other amyloid-like films prepared from lysozyme, insulin, and α-lactalbumin^49^. Although lysozyme amyloid fibrils, compared to their monomer, exhibit better antibacterial activities against both *Staphylococcus aureus* and *Escherichia coli* bacteria^100^, to endow a complete antifouling surface further modification is still desirable. To this end, poly(ethylene glycol) (PEG) can be conjugated with amyloid-like aggregation to form PEG coated-amyloid nanofilm. The resultant nanofilm’s antifouling properties were assessed by a series of solutes, including proteins, biofluids, components of extracellular polymeric substances, carbohydrates and polysaccharides, esters, and bacteria, demonstrating improved antifouling properties^101^. Similarly, polylysine (ε-PL) was immobilized on the BSA amyloid-like film to improve antifouling and provide antimicrobial features^102^. Another method for controlling fouling involves micro-nanopatterned surface topographies by employing a soft-lithography approach to realize micro and nanostructured silk films with different geometries. Compared to the flat control, the patterned silk film exhibited better antifouling properties, by a 66% reduction in the number of adhered bacteria to the surface^64^. These works demonstrate that the antifouling of protein nanofibrils membranes may be achieved by several possible strategies.

The structural stability of protein nanofibrils during water purification in acidic or alkaline conditions is another important parameter that should be considered. Typically, most heavy metals wastewater is in acidic condition. Since amyloid fibrils are prepared via unfolding, hydrolysis and aggregation in strongly acidic conditions, they are ideally suited to this application, as their structure and functionality remain excellent and stable under these conditions^22^; yet their performance is preserved up to very basic pHs^8^. Silk nanofibrils have been widely used for removing heavy metals, and no evidence of their degradation in acidic conditions so far is reported^31,33^. However, degradation of silk nanofibrils under extended servicing at acidic conditions, especially in presence of sulfuric acid, cannot be neglected since complex hydrolysis processes can indeed occur^103^.

In this review, we have highlighted how the fully sustainable footprint of protein nanofibrils water purification agents places them at the cutting-edge of modern technologies in water purification. We have benchmarked their sustainability profile against established adsorbents in water purification, such as activated carbon and nanocellulose and demonstrated via LCA the benefits of protein nanofibrils water purification agents over other bio-adsorbents. Protein nanofibrils possess some unique technical key properties ideally suited to excel in water purification, such as their high surface-to-volume ratio coupled with the broad range of amino acids present on their surface, which endow these systems characteristics similar to multiple exchange resins, yet combined together within a single, natural system. Additionally, they offer the possibility to design adsorbing materials made thereof in a multitude of shapes serving a wide range of application requirements and boundary conditions. As a result of the unique combination of their origin, source, performance, cost, and ease of implementation, protein nanofibrils are emerging as an unchallenged response to the pressing problem of sustainable water purification. It is anticipated that this affordable and green set of technologies can resolve—or at least mitigate—the pressing global water crisis and improves the life quality of millions of people around the world, meeting this challenge in a fully sustainable way.

## Supplementary information

Supplementary Information

## References

[1] United Nations. *Transforming Our World: the 2030 Agenda for Sustainable Development A/RES/70/1* (UN General Assembly, 2015).

[2] Stockholm Environment Institute, 6 Clean Water and Sanitation, The Government of Sweden. https://www.government.se/49f47b/contentassets/3bef47b49ed64a75bcdf56ff053ccaea/6---clean-water-and-sanitation.pdf.

[3] United Nations Children’s Fund (UNICEF) and World Health Organization. *Progress on Household Drinking Water, Sanitation and Hygiene 2000-2017. Special Focus on Inequalities* (United Nations Children’s Fund (UNICEF) and World Health Organization, 2019).

[4] Bolisetty S, Peydayesh M, Mezzenga R (2019). Sustainable technologies for water purification from heavy metals: review and analysis. Chem. Soc. Rev..

[5] Ibrahim Y, Arafat HA, Mezher T, AlMarzooqi F (2018). An integrated framework for sustainability assessment of seawater desalination. Desalination.

[6] Stewart RJ, Ransom TC, Hlady V (2011). Natural underwater adhesives. J. Polym. Sci. B Polym. Phys..

[7] Faltova L, Küffner AM, Hondele M, Weis K, Arosio P (2018). Multifunctional protein materials and microreactors using low complexity domains as molecular adhesives. ACS Nano.

[8] Bolisetty S, Mezzenga R (2016). Amyloid–carbon hybrid membranes for universal water purification. Nat. Nanotechnol..

[9] Cao Y, Mezzenga R (2019). Food protein amyloid fibrils: origin, structure, formation, characterization, applications and health implications. Adv. Colloid Interface Sci..

[10] Food and Agriculture Organization of the United Nations. *Save Food: Global Initiative on Food Loss and Waste Reduction* (FAO, 2016).

[11] Mahboubi A, Ferreira JA, Taherzadeh MJ, Lennartsson PR (2017). Value-added products from dairy waste using edible fungi. Waste Manag..

[12] Sá AGA, Moreno YMF, Carciofi BAM (2020). Plant proteins as high-quality nutritional source for human diet. Trends Food Sci. Technol..

[13] Ling S, Kaplan DL, Buehler MJ (2018). Nanofibrils in nature and materials engineering. Nat. Rev. Mater..

[14] Ye, X., Lendel, C., Langton, M., Olsson, R. T. & Hedenqvist, M. S. Protein nanofibrils: Preparation, properties, and possible applications in industrial nanomaterials. In *Industrial Applications of Nanomaterials* (eds Sabu, T., Yves G. & Yasir B. P.) 29–63 (Elsevier, 2019).

[15] Ling S (2014). Modulating materials by orthogonally oriented β-strands: composites of amyloid and silk fibroin fibrils. Adv. Mater..

[16] Knowles TPJ, Mezzenga R (2016). Amyloid fibrils as building blocks for natural and artificial functional materials. Adv. Mater..

[17] Wei G (2017). Self-assembling peptide and protein amyloids: from structure to tailored function in nanotechnology. Chem. Soc. Rev..

[18] Peydayesh M (2020). Amyloid fibrils aerogel for sustainable removal of organic contaminants from water. Adv. Mater..

[19] Lambrecht MA (2019). Conditions governing food protein amyloid fibril formation. part II: milk and legume proteins. Compr. Rev. Food Sci. Food Saf..

[20] Ling S, Jin K, Kaplan DL, Buehler MJ (2016). Ultrathin free-standing Bombyx mori silk nanofibril membranes. Nano Lett..

[21] Han X (2019). Sheet-like and tubular aggregates of protein nanofibril–phosphate hybrids. Chem. Commun..

[22] Peydayesh M, Bolisetty S, Mohammadi T, Mezzenga R (2019). Assessing the binding performance of amyloid-carbon membranes toward heavy metal ions. Langmuir.

[23] van Lith, R. & Ameer, G. A. Antioxidant Polymers as Biomaterial. In *Oxidative Stress and Biomaterials* (eds Thomas D. & D. Allan Butterfield) 251–296 (Academic Press, 2016).

[24] Sudan RJJ, Kumari JLJ, Sudandiradoss C (2015). Ab initio coordination chemistry for nickel chelation motifs. PLoS ONE.

[25] Zhao J, Yang P (2020). Amyloid-mediated fabrication of organic–inorganic hybrid materials and their biomedical applications. Adv. Mater. Interfaces.

[26] Yang F, Tao F, Li C, Gao L, Yang P (2018). Self-assembled membrane composed of amyloid-like proteins for efficient size-selective molecular separation and dialysis. Nat. Commun..

[27] Yang F (2020). Rapid capture of trace precious metals by amyloid-like protein membrane with high adsorption capacity and selectivity. J. Mater. Chem. A.

[28] Reynolds NP, Charnley M, Mezzenga R, Hartley PG (2014). Engineered lysozyme amyloid fibril networks support cellular growth and spreading. Biomacromolecules.

[29] Wu X (2018). Supramolecular proteinaceous biofilms as trapping sponges for biologic water treatment and durable catalysis. J. Colloid Interface Sci..

[30] Ling S, Li C, Jin K, Kaplan DL, Buehler MJ (2016). Liquid exfoliated natural silk nanofibrils: applications in optical and electrical devices. Adv. Mater..

[31] Ling S (2017). Design and function of biomimetic multilayer water purification membranes. Sci. Adv..

[32] Zhang Q (2019). Selective and efficient removal of fluoride from water: in situ engineered amyloid Fibril/ZrO2 hybrid membranes. Angew. Chem. Int. Ed..

[33] Zhao F, Peydayesh M, Ying Y, Mezzenga R, Ping J (2020). Transition metal dichalcogenide–silk nanofibril membrane for one-step water purification and precious metal recovery. ACS Appl. Mater. Interfaces.

[34] Bolisetty, S., Coray, N. M., Palika, A., Prenosil, G. A. & Mezzenga, R. Amyloid hybrid membranes for removal of clinical and nuclear radioactive wastewater. *Environ. Sci. Water Res. Technol*. 10.1039/d0ew00693a (2020).

[35] Heyn TR (2020). The threshold of amyloid aggregation of beta-lactoglobulin: relevant factor combinations. J. Food Eng..

[36] Peydayesh M, Pauchard M, Bolisetty S, Stellacci F, Mezzenga R (2019). Ubiquitous aluminium contamination in water and amyloid hybrid membranes as a sustainable possible solution. Chem. Commun..

[37] Bolisetty S, Reinhold N, Zeder C, Orozco MN, Mezzenga R (2017). Efficient purification of arsenic-contaminated water using amyloid–carbon hybrid membranes. Chem. Commun..

[38] Dražević E, Košutić K, Freger V (2014). Permeability and selectivity of reverse osmosis membranes: correlation to swelling revisited. Water Res..

[39] Ramírez-Rodríguez LC (2020). Preparation of a hybrid membrane from whey protein fibrils and activated carbon to remove mercury and chromium from water. Membranes.

[40] Wu T (2019). What is new in lysozyme research and its application in food industry? A review. Food Chem..

[41] Shahmohammadi A (2018). Lysozyme separation from chicken egg white: a review. Eur. Food Res. Technol..

[42] Lesnierowski G, Stangierski J (2018). What’s new in chicken egg research and technology for human health promotion? - A review. Trends Food Sci. Technol..

[43] Morshedi D, Mohammadi Z, Akbar Boojar MM, Aliakbari F (2013). Using protein nanofibrils to remove azo dyes from aqueous solution by the coagulation process. Colloids Surf. B Biointerfaces.

[44] Leung W-H, Lo W-H, Chan P-H (2015). Amyloid fibrils as rapid and efficient nano-biosorbents for removal of dye pollutants. RSC Adv..

[45] Silva NHCS (2020). Dual nanofibrillar-based bio-sorbent films composed of nanocellulose and lysozyme nanofibrils for mercury removal from spring waters. Carbohydr. Polym..

[46] Liu M (2020). Fast and robust lead (II) removal from water by bioinspired amyloid lysozyme fibrils conjugated with polyethyleneimine (PEI). Chem. Eng. J..

[47] Leung W-H, So P-K, Wong W-T, Lo W-H, Chan P-H (2016). Ethylenediamine-modified amyloid fibrils of hen lysozyme with stronger adsorption capacity as rapid nano-biosorbents for removal of chromium(vi) ions. RSC Adv..

[48] Liu, R. et al. Aggregation-induced emission of a 2D protein supramolecular nanofilm with emergent functions. *Mater. Chem. Front*. 10.1039/D0QM00031K (2020).

[49] Hu X (2020). Amyloid-like protein aggregates: a new class of bioinspired materials merging an interfacial anchor with antifouling. Adv. Mater..

[50] Aramwit, P. Introduction to biomaterials for wound healing. In *Wound Healing Biomaterials* (ed Magnus, S. Å.) 3–38 (Woodhead Publishing, 2016).

[51] Ramya R, Muthukumaran P, Wilson J (2018). Electron beam-irradiated polypyrrole decorated with bovine serum albumin pores: simultaneous determination of epinephrine and L-tyrosine. Biosens. Bioelectron..

[52] Jaglińska K (2020). Retardation of some drugs in thin-layer chromatographic systems with impregnated silica gel plates with hen’s egg white and bovine serum albumin. J. Chromatogr. A.

[53] Yu X, Liu W, Deng X, Yan S, Su Z (2018). Gold nanocluster embedded bovine serum albumin nanofibers-graphene hybrid membranes for the efficient detection and separation of mercury ion. Chem. Eng. J..

[54] Zhu Y, Yu X, Zhang T, Li P, Wang X (2020). Biomimetic sulfated silk nanofibrils for constructing rapid mid-molecule toxins removal nanochannels. J. Membr. Sci..

[55] Yi S (2018). Scalable fabrication of sulfated silk fibroin nanofibrous membranes for efficient lipase adsorption and recovery. Int. J. Biol. Macromol..

[56] Godiya CB, Cheng X, Deng G, Li D, Lu X (2019). Silk fibroin/polyethylenimine functional hydrogel for metal ion adsorption and upcycling utilization. J. Environ. Chem. Eng..

[57] Wang S (2019). Preparation and characterization of graphene oxide/silk fibroin hybrid aerogel for dye and heavy metal adsorption. Compos. B. Eng..

[58] Yi S (2017). Ultrafine silk-derived nanofibrous membranes exhibiting effective lysozyme adsorption. ACS Sustain. Chem. Eng..

[59] Chen T, Duan M, Shi P, Fang S (2017). Ultrathin nanoporous membranes derived from protein-based nanospheres for high-performance smart molecular filtration. J. Mater. Chem. A.

[60] Xiong R (2017). Template-guided assembly of silk fibroin on cellulose nanofibers for robust nanostructures with ultrafast water transport. ACS Nano.

[61] P A (2017). Removal of toxic heavy metal lead (II) using chitosan oligosaccharide-graft-maleic anhydride/polyvinyl alcohol/silk fibroin composite. Int. J. Biol. Macromol..

[62] Gao A, Xie K, Song X, Zhang K, Hou A (2017). Removal of the heavy metal ions from aqueous solution using modified natural biomaterial membrane based on silk fibroin. Ecol. Eng..

[63] Li Z, Tan CM, Tio W, Ang J, Sun DD (2018). Manta ray gill inspired radially distributed nanofibrous membrane for efficient and continuous oil–water separation. Environ. Sci. Nano.

[64] Tullii G (2020). Micro- and nanopatterned silk substrates for antifouling applications. ACS Appl. Mater. Interfaces.

[65] Li C (2014). Amyloid-hydroxyapatite bone biomimetic composites. Adv. Mater..

[66] Li Z (2018). Biomineralization-mimetic preparation of hybrid membranes with ultra-high loading of pristine metal–organic frameworks grown on silk nanofibers for hazard collection in water. J. Mater. Chem. A.

[67] Lv L (2017). Biomimetic hybridization of kevlar into silk fibroin: nanofibrous strategy for improved mechanic properties of flexible composites and filtration membranes. ACS Nano.

[68] Kiseleva, A. P., Krivoshapkin, P. V. & Krivoshapkina, E. F. Recent advances in development of functional spider silk-based hybrid materials. *Front. Chem*. 10.3389/fchem.2020.00554 (2020). 10.3389/fchem.2020.00554 PMC7338834 32695749

[69] Pelit L, Ertaş FN, Eroğlu AE, Shahwan T, Tural H (2011). Biosorption of Cu(II) and Pb(II) ions from aqueous solution by natural spider silk. Bioresour. Technol..

[70] Krishnaji ST, Kaplan DL (2013). Bioengineered chimeric spider silk-uranium binding proteins. Macromol. Biosci..

[71] Rachwał M, Rybak J, Rogula-Kozłowska W (2018). Magnetic susceptibility of spider webs as a proxy of airborne metal pollution. Environ. Pollut..

[72] Xiao-li S, Yu P, Hose GC, Jian C, Feng-xiang L (2006). Spider webs as indicators of heavy metal pollution in air. Bull. Environ. Contamination Toxicol..

[73] Ang WL, Mohammad AW (2020). State of the art and sustainability of natural coagulants in water and wastewater treatment. J. Clean. Prod..

[74] Kamali M, Suhas DP, Costa ME, Capela I, Aminabhavi TM (2019). Sustainability considerations in membrane-based technologies for industrial effluents treatment. Chem. Eng. J..

[75] Palmer Timothy B, Flanagan David J (2016). The sustainable company: looking at goals for people, planet and profits. J. Bus. Strategy.

[76] Roy S (2020). Evaluating strategies for environmental sustainability in a supply chain of an emerging economy. J. Clean. Prod..

[77] Cariola A, Fasano F, La Rocca M, Skatova E (2020). Environmental sustainability policies and the value of debt in EU SMEs: empirical evidence from the energy sector. J. Clean. Prod..

[78] Lawler W (2012). Towards new opportunities for reuse, recycling and disposal of used reverse osmosis membranes. Desalination.

[79] Szekely G, Jimenez-Solomon MF, Marchetti P, Kim JF, Livingston AG (2014). Sustainability assessment of organic solvent nanofiltration: from fabrication to application. Green. Chem..

[80] Jabbour, R., Salem, H. & Sidell, F. R. Blister Agents/Vesicants. In *Encyclopedia of Toxicology* 3rd edn (ed. Philip, W.) 522–525 (Academic Press, 2014).

[81] Petruccioli, M., Raviv, M., Di Silvestro, R. & Dinelli, G. Agriculture and Agro-Industrial Wastes, Byproducts, and Wastewaters: Origin, Characteristics, and Potential in Bio-Based-Compounds Production. In *Comprehensive Biotechnology* 2nd edn (ed Murray, M.-Y.) 531–545 (Academic Press, 2011).

[82] Cornejo PK, Santana MVE, Hokanson DR, Mihelcic JR, Zhang Q (2014). Carbon footprint of water reuse and desalination: a review of greenhouse gas emissions and estimation tools. J. Water Reuse Desalination.

[83] Gadalla MA, Olujic Z, Jansens PJ, Jobson M, Smith R (2005). Reducing CO2 emissions and energy consumption of heat-integrated distillation systems. Environ. Sci. Technol..

[84] Dunmade I (2002). Indicators of sustainability: assessing the suitability of a foreign technology for a developing economy. Technol. Soc..

[85] Popovic, T., Kraslawski, A., Heiduschke, R. & Repke, J.-U. Indicators of Social Sustainability for Wastewater Treatment Processes. In *Computer Aided Chemical Engineering* Vol. 34 (eds Mario, R. E., John, D. S. & Gavin, P. T.) 723–728 (Elsevier, 2014).

[86] United Nations. *The Sustainable Development Goals Report 2020* (United Nations, 2020).

[87] FAO. *The 10 Elements of Agroecology, Guiding the Transition to Sustainable Food and Agricultural Systems* (FAO, 2018).

[88] Thompson KA (2016). Environmental comparison of biochar and activated carbon for tertiary wastewater treatment. Environ. Sci. Technol..

[89] Bayer P, Heuer E, Karl U, Finkel M (2005). Economical and ecological comparison of granular activated carbon (GAC) adsorber refill strategies. Water Res..

[90] Toczko, J. F. Catalyst Recovery and Recycle: Metal Removal Techniques. In *Comprehensive Chirality* (eds Erick, M. C. & Hisashi Y.) (Elsevier, 2012).

[91] Backer, H. in *Travel Medicine* 4th edn (eds Jay, S. K. et al.) 209–227 (Elsevier, 2019).

[92] Mahfoudhi N, Boufi S (2017). Nanocellulose as a novel nanostructured adsorbent for environmental remediation: a review. Cellulose.

[93] Voisin H, Bergström L, Liu P, Mathew AP (2017). Nanocellulose-based materials for water purification. Nanomaterials.

[94] Walser T, Demou E, Lang DJ, Hellweg S (2011). Prospective environmental life cycle assessment of nanosilver T-shirts. Environ. Sci. Technol..

[95] Arvidsson R, Kushnir D, Sandén BA, Molander S (2014). Prospective life cycle assessment of graphene production by ultrasonication and chemical reduction. Environ. Sci. Technol..

[96] Kazemi A, Bahramifar N, Heydari A, Olsen SI (2018). Life cycle assessment of nanoadsorbents at early stage technological development. J. Clean. Prod..

[97] Yang R (2014). Thiol-modified cellulose nanofibrous composite membranes for chromium (VI) and lead (II) adsorption. Polymer.

[98] Li Q, McGinnis S, Sydnor C, Wong A, Renneckar S (2013). Nanocellulose life cycle assessment. ACS Sustain. Chem. Eng..

[99] Arvidsson R, Nguyen D, Svanström M (2015). Life cycle assessment of cellulose nanofibrils production by mechanical treatment and two different pretreatment processes. Environ. Sci. Technol..

[100] Wei Z (2021). Enhanced antibacterial activity of hen egg-white lysozyme against Staphylococcus aureus and Escherichia coli due to protein fibrillation. Biomacromolecules.

[101] Li C, Lu D, Deng J, Zhang X, Yang P (2019). Amyloid-like rapid surface modification for antifouling and in-depth remineralization of dentine tubules to treat dental hypersensitivity. Adv. Mater..

[102] Tian J (2020). Amyloid-like protein aggregates combining antifouling with antibacterial activity. Biomater. Sci..

[103] Hu Y, Yu J, Liu L, Fan Y (2019). Preparation of natural amphoteric silk nanofibers by acid hydrolysis. J. Mater. Chem. B.

[104] Carpenter AW, de Lannoy C-F, Wiesner MR (2015). Cellulose nanomaterials in water treatment technologies. Environ. Sci. Technol..

[105] Mittal N (2017). Ultrastrong and bioactive nanostructured bio-based composites. ACS Nano.

[106] Schwarzenbach RP, Egli T, Hofstetter TB, Gunten UV, Wehrli B (2010). Global water pollution and human health. Annu. Rev. Environ. Resour..

