# Supplementary material for: Protein nanofibrils for next generation sustainable water purification
Source: Nat Commun. 2021 May 31;12:3248. doi: 10.1038/s41467-021-23388-2 (PMC8166862; doi:10.1038/s41467-021-23388-2)
Supplement: Supplementary file 1 — Supplementary Information [file 41467_2021_23388_MOESM1_ESM.pdf]

Supplementary Information

Protein Nanofibrils for Next Generation Sustainable Water Purification

Mohammad Peydayesh[1] and Raffaele Mezzenga[1, 2,*]

[1]ETH Zurich, Department of Health Sciences and Technology, 8092 Zurich, Switzerland

[2]ETH Zurich, Department of Materials, 8093 Zurich, Switzerland

E-mail: raffaele.mezzenga@hest.ethz.ch

**Supplementary Table 1 | Sustainability assessment of protein nanofibrils water purification agents based on the three main sustainability pillars**	
**Sustainability pillars**	**Description**
**1. Environmental**	
Material eco-compatibility and life cycle	Protein nanofibrils are natural-based, unlike synthetic materials such as plastics and polymers. They are entirely biodegradable and environmentally friendly.
Secondary pollution	There is no release of potentially harmful chemical substances from the nanofibrils water purification agents. Unlike degradation technologies, there is no metabolite generation, which could cause secondary pollution.
Potential to recover valuable heavy metals	Protein nanofibrils offer the possibility to reduce and recover the adsorbed contaminants such as heavy and precious metals, increasing their value as a sustainable water purification agent.
Waste and byproduct use and management	In many cases, the source of protein nanofibrils can be from industrial and agricultural waste and byproducts. Waste management by utilizing it and minimizing its disposal in environment is a key advantage of water purification by protein nanofibrils.
Carbon footprint	Differently from other energy-intensive technologies, protein nanofibrils water purification agents can operate under gravity-driven filtration, minimizing the energy consumption and CO_2 emission.
**2. Techno-economic**	
Treatment efficiency	Protein nanofibrils water purification agents have excellent removal efficiencies for a broad-spectrum of contaminants such as heavy metals, metalloids, radioactive wastes, pesticides, dyes, pharmaceuticals and phenolic compounds. Compared to the typical water treatment techniques, they offer faster binding and higher flux rates.
Product stability and reliability	Protein nanofibrils water purification agents are reliable and stable during the long-term operation and under different effluent conditions. In some cases (e.g. membranes and aerogels) they can be easily regenerated and reused in the process without significant changes in performance.
Safety	Thanks to their natural origin, protein nanofibrils are non-toxic and minimize health and safety risks regarding their application.

Ease of implementation and scale-up	Protein nanofibrils are suitable for a multitude of plant facilities, including membranes, cartridges, adsorption columns, granular sedimentation ponds, etc., from industrial to household levels due to their design flexibility, ease of operation and low maintenance needs.
Compatibility with other technologies	Protein nanofibrils water purification agents can be easily combined and integrated with other techniques to optimize the water purification process in sequential step treatments.
Cost of production and operation	Due to their origin and provenance, protein nanofibrils can be extremely affordable. The purification process from waste and byproduct sources can be carried out very inexpensively. Operating costs are minimal, and require virtually no energy, for example, by vacuum filtration and/or gravity driving forces.
Impact on economy	Protein nanofibrils technologies add value within the economy by elevating a waste product from industry to a high commodity product and technological material for water purification.
**3. Social**	
Public acceptance	The possibility to purify water by reducing food waste at a virtually zero CO_2 emission and zero energy consumption make protein nanofibrils an outstanding technology from public acceptance perspective, with an extremely low environmental footprint and exceptional performance.
Improvement of public health and life quality	By globally providing clean water via a natural, simple and cheap approach, protein nanofibrils have a bright future ahead and could play a pivotal role in clean water supplying worldwide. Clean water consumption improves public health and the quality of life in general.
Sustainable Development Goals (UN)	Water purification using protein nanofibrils meets several of the sustainable development goals set by the United Nations for the 2030 agenda.

**Supplementary Table 2 | Life cycle inventory data**						
**1. Whey amyloid fibrils**						
Whey powder production						
Process Inputs						
	Chemicals	Quantity	Units	SimaPro Process	Data base	Notes
Material	Whey	31.25	g	Whey {GLO}| market for | Alloc Def	EcoInvent	Liquid whey 6%
	Equipment	Quantity	Units	SimaPro Process	Data base	Notes
Electricity	*Liquid Whey to powder whey*					
	Electricity	0.0024	kW.h	Electricity, high voltage {GLO}| market group for | Alloc Def	EcoInvent	1.2kWh electricity per kg of Whey[1]
	Heating and stirring plate	0.01088	kW.h	Heat, district or industrial, natural gas {Europe without Switzerland}| market for heat, district or industrial, natural gas | Alloc Def	EcoInvent	5.44kWh thermal energy per kg of Whey[1]
Process Outputs						
	Chemicals	Quantity	Units	SimaPro Process	Data base	Notes
	Whey powder monomer	2	g	N/A	N/A	
Fibrilization						
Process Inputs						
	Chemicals	Quantity	Units	SimaPro Process	Data base	Notes
Material	Whey protein monomer	2	g			
	HCl	0.275	ml	Hydrochloric acid, Mannheim process (30% HCl), at plant/RER Economic	EcoInvent	
	Water for reaction	0.1	Liter	Water, deionised, from tap water, at user {CH}| production | Alloc Def	EcoInvent	
	Equipment	Quantity	Units	SimaPro Process	Data base	Notes
Electricity	Heating and stirring plate	0.585	kW.h	Electricity, high voltage {GLO}| market group for | Alloc Def	EcoInvent	
Process Outputs						

	Chemicals	Quantity	Units	SimaPro Process	Data base	Notes
	Whey Amyloid fibrils	2	g	N/A	N/A	
**2. TEMPO nanocellulose**[2]						
Process Inputs						
	Chemicals	Quantity	Units	SimaPro Process	Data base	Notes
Material	Kraft wood pulp	40	g	Sulfate pulp [3]| production, elementary chlorine free bleached | Alloc Def	EcoInvent	Dry mass ~25%
	TEMPO	0.06	g	N/A	N/A	Empty process; no data
	NaClO	21.4 (or 19.28 g)	ml	Sodium hypochlorite, without water, in 15% solution state {GLO}| market for | Alloc Def	EcoInvent	
	NaBr	2.4	g	N/A	N/A	Empty process; no data - replaced by NaCl
	NaOH	50 (or 4 g)	ml	Neutralising agent, sodium hydroxide-equivalent {GLO}| market for | Alloc Def	EcoInvent	
	Ethanol	200 (or 250 g)	ml	Ethanol, without water, in 99.7% solution state, from fermentation {GLO}| market for | Alloc Def	EcoInvent	
	NaCl	1.35	g	Sodium chloride, powder {GLO}| market for | Alloc Def	EcoInvent	To model NaBr
	Water for reaction	0.5	Liter	Water, deionised, from tap water, at user {CH}| production | Alloc Def	EcoInvent	
	Water for washing	5	Liter	Water, deionised, from tap water, at user {CH}| production | Alloc Def	EcoInvent	
	Equipment	Quantity	Units	SimaPro Process	Data base	Notes
Electricity	Cable blender	0.055	kW.h	Electricity, high voltage {GLO}| market group for | Alloc Def	EcoInvent	
	Syringe pump	0.01	kW.h	Electricity, high voltage {GLO}| market group for | Alloc Def	EcoInvent	

	Centrifuge washer	0.07	kW.h	Electricity, high voltage {GLO}| market group for | Alloc Def	EcoInvent	
Process Outputs						
	Chemicals	Quantity	Units	SimaPro Process	Data base	Notes
	Chemically altered cellulose	10	g	N/A	N/A	
Hemogenization						
Process Inputs						
	Chemicals	Quantity	Units	SimaPro Process	Data base	Notes
Product	Chemically altered cellulose	10	g	N/A	N/A	
	Equipment	Quantity	Units	SimaPro Process	Data base	Notes
Electricity	Hemogenizer	0.96	kW.h	Electricity, high voltage {GLO}| market group for | Alloc Def	EcoInvent	
Process Outputs						
	Chemicals	Quantity	Units	SimaPro Process	Data base	Notes
	Chemically altered cellulose	10	g	N/A	N/A	
3. **Activated carbon**						
Process Inputs						
	Chemicals	Quantity	Units	SimaPro Process	Data base	Notes
Material	Activated carbon	1	g	Activated carbon, granular {GLO}| market for activated carbon, granular | Alloc Def	EcoInvent	
Process Outputs						
	Chemicals	Quantity	Units	SimaPro Process	Data base	Notes

	Activated carbon	1	g	Activated carbon, granular {GLO}| market for activated carbon, granular | Alloc Def	EcoInvent	

Supplementary Table 3 | LCA impact assessment based on ReCiPe Midpoint method

**Impact category**	**Unit**	**Activated Carbon**	**Protein nanofibrils**	**TEMPO-Nanocellulose**
Climate change	kg CO_2 eq	2994.2554	223.7118	1942.2011
Ozone depletion	kg CFC-11 eq	0.0001	0.0000	0.0001
Terrestrial acidification	kg SO_2 eq	16.6856	1.0316	10.6541
Freshwater eutrophication	kg P eq	1.0972	0.1160	0.8381
Marine eutrophication	kg N eq	0.4736	0.0562	2.2821
Human toxicity	kg 1,4-DB eq	807.7014	75.2448	643.4112
Photochemical oxidant formation	kg NMVOC	7.4774	0.5566	8.4148
Particulate matter formation	kg PM10 eq	6.1854	0.7016	6.2606
Terrestrial ecotoxicity	kg 1,4-DB eq	0.0772	0.0220	12.2240
Freshwater ecotoxicity	kg 1,4-DB eq	20.3437	1.9317	16.6412
Marine ecotoxicity	kg 1,4-DB eq	19.0453	1.8051	14.3285
Ionising radiation	kBq U235 eq	131.6392	31.3632	212.5412
Agricultural land occupation	m^2a	71.7286	13.0646	1148.6474
Urban land occupation	m^2a	20.5084	1.2902	36.0135
Natural land transformation	m^2	0.2293	0.0337	7.1546
Water depletion	m^3	7.5065	1.7828	111.4294
Metal depletion	kg Fe eq	16.1920	2.1401	36.3264
Fossil depletion	kg oil eq	767.4591	57.5218	417.9915

Supplementary References

1 González-García, S., Hospido, A., Moreira, M. T., Feijoo, G. & Arroja, L. Environmental Life Cycle Assessment of a Galician cheese: San Simon da Costa. *Journal of Cleaner Production* **52**, 253-262, doi:https://doi.org/10.1016/j.jclepro.2013.03.006 (2013).

2 Li, Q., McGinnis, S., Sydnor, C., Wong, A. & Renneckar, S. Nanocellulose Life Cycle Assessment. *ACS Sustainable Chemistry & Engineering* **1**, 919-928, doi:10.1021/sc4000225 (2013).

3 Sanchez-Garcia, L. *et al.* Recombinant pharmaceuticals from microbial cells: a 2015 update. *Microbial Cell Factories* **15**, 33, doi:10.1186/s12934-016-0437-3 (2016).
